# Measurement bias in caregiver‐report of early childhood behavior problems across demographic factors in an ECHO‐wide diverse sample

**DOI:** 10.1002/jcv2.12198

**Published:** 2023-09-20

**Authors:** Shuting Zheng, Maxwell Mansolf, Monica McGrath, Marie L. Churchill, Traci A. Bekelman, Patricia A. Brennan, Amy E. Margolis, Sara S. Nozadi, Theresa M. Bastain, Amy J. Elliott, Kaja Z. LeWinn, Julie A. Hofheimer, Leslie D. Leve, Brandon Rennie, Emily Zimmerman, Carmen A. Marable, Cindy T. McEvoy, Chang Liu, Alexis Sullivan, Tracey J. Woodruff, Samiran Ghosh, Bennett Leventhal, Assiamira Ferrara, Johnnye Lewis, Somer Bishop, Environmental influences on Child Health Outcomes

**Affiliations:** ^1^ Department of Psychiatry and Behavioral Sciences University of California San Francisco CA USA; ^2^ Department of Medical Social Sciences Feinberg School of Medicine Northwestern University Chicago IL USA; ^3^ Department of Epidemiology Johns Hopkins Bloomberg School of Public Health Baltimore MD USA; ^4^ Department of Epidemiology Colorado School of Public Health Aurora CO USA; ^5^ Department of Psychology Emory University Atlanta GA USA; ^6^ Department of Psychiatry Columbia University Irving Medical Center New York State Psychiatric Institute New York NY USA; ^7^ Community Environmental Health College of Pharmacy Health Sciences Center University of New Mexico Albuquerque NM USA; ^8^ Department of Population and Public Health Sciences University of Southern California Los Angeles CA USA; ^9^ Avera Research Institute Sioux Falls SD USA; ^10^ Department of Pediatrics Division of Neonatal‐Perinatal Medicine North Carolina at Chapel Hill Chapel Hill NC USA; ^11^ Prevention Science Institute University of Oregon Eugene OR USA; ^12^ Health Sciences Center Department of Pediatrics Center for Development and Disability University of New Mexico Navajo Birth Cohort Study Albuquerque NM USA; ^13^ Communication Sciences & Disorders Northeastern University Boston MA USA; ^14^ School of Medicine University of North Carolina at Chapel Hill Neuroscience Curriculum Chapel Hill NC USA; ^15^ Department of Pediatrics Pape Pediatric Research Institute Oregon Health & Science University Portland OR USA; ^16^ Department of Psychology Washington State University Pullman WA USA; ^17^ Center for Health and Community University of California San Francisco CA USA; ^18^ Department of Biostatistics and Data Science & Coordinating Center for Clinical Trials (CCCT) University of Texas School of Public Health Houston TX USA; ^19^ University of Chicago Navajo Birth Cohort Study University of New Mexico Albuquerque NM USA; ^20^ Division of Research Kaiser Permanente Northern California Oakland CA USA; ^21^ Navajo Birth Cohort Study Community Environmental Health Program College of Pharmacy University of New Mexico Albuquerque NM USA; ^22^ Department of Psychiatry and Behavioral Sciences Weill Institute for Neurosciences University of California San Francisco CA USA

**Keywords:** behavior problems, behavioral measures, pre‐school children, psychometrics

## Abstract

**Background:**

Research and clinical practice rely heavily on caregiver‐report measures, such as the Child Behavior Checklist 1.5–5 (CBCL/1.5‐5), to gather information about early childhood behavior problems and to screen for child psychopathology. While studies have shown that demographic variables influence caregiver ratings of behavior problems, the extent to which the CBCL/1.5‐5 functions equivalently at the item level across diverse samples is unknown.

**Methods:**

Item‐level data of CBCL/1.5‐5 from a large sample of young children (*N* = 9087) were drawn from 26 cohorts in the Environmental influences on Child Health Outcomes program. Factor analyses and the alignment method were applied to examine measurement invariance (MI) and differential item functioning (DIF) across child (age, sex, bilingual status, and neurodevelopmental disorders), and caregiver (sex, education level, household income level, depression, and language version administered) characteristics. Child race was examined in sensitivity analyses.

**Results:**

Items with the most impactful DIF across child and caregiver groupings were identified for Internalizing, Externalizing, and Total Problems. The robust item sets, excluding the high DIF items, showed good reliability and high correlation with the original Internalizing and Total Problems scales, with lower reliability for Externalizing. Language version of CBCL administration, education level and sex of the caregiver respondent showed the most significant impact on MI, followed by child age. Sensitivity analyses revealed that child race has a unique impact on DIF over and above socioeconomic status.

**Conclusions:**

The CBCL/1.5‐5, a caregiver‐report measure of early childhood behavior problems, showed bias across demographic groups. Robust item sets with less DIF can measure Internalizing and Total Problems equally as well as the full item sets, with slightly lower reliability for Externalizing, and can be crosswalked to the metric of the full item set, enabling calculation of normed T scores based on more robust item sets.

## Graphical abstract

This study systematically examines the measurement bias of the CBCL 1.5‐5 in a large, diverse sample (n = 9087). Questions from a commonly used measure of behavior problems in young children show bias depending on the language version administered (Spanish vs. English), educational level and sex of the caregiver respondent, as well as child’s age. Findings suggest that this questionnaire might not measure behavior problems in the same way for children with caregivers from diverse backgrounds. Recommendations are provided for bias‐reduced item sets with scores mapped onto the original scale.

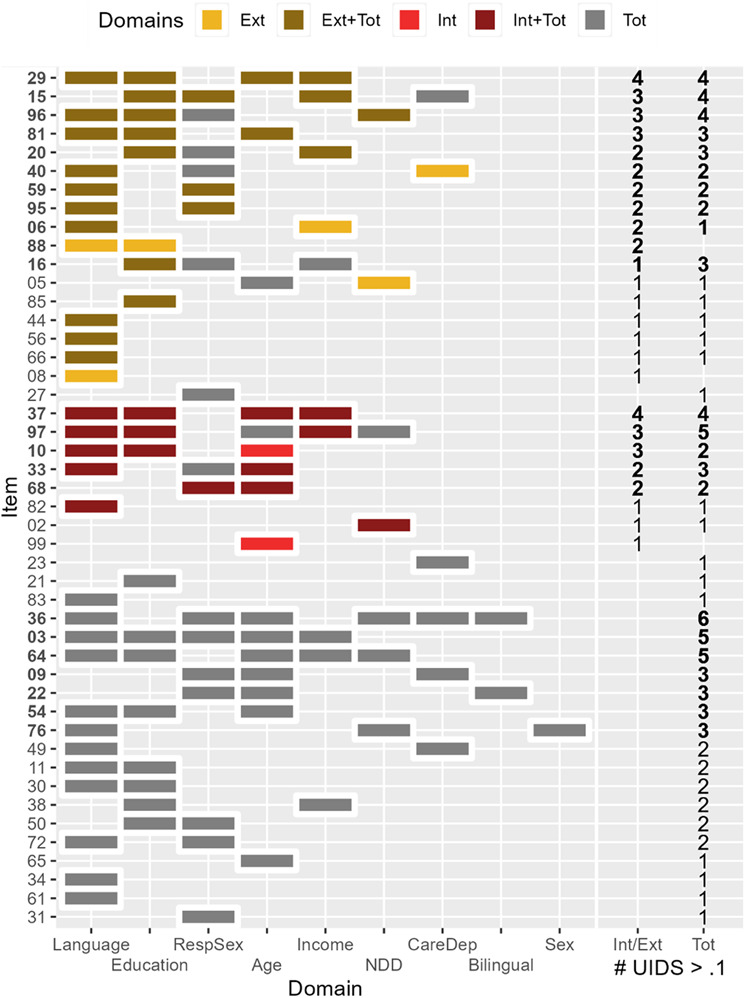

**Key points**

- Caregiver characteristics, especially language version, education level and sex of the caregiver respondent, greatly impacted the differential functioning of many CBCL/1.5‐5 items.
- Child age also influenced the measurement of child behavior problems on the CBCL/1.5‐5.
- SES variables (i.e., caregiver education and household income levels) cannot fully account for measurement bias related to child race.
- Robust item sets with less DIF can reliably capture Internalizing, Externalizing, and Total Problems with less measurement bias

## INTRODUCTION

Behavior problems, including noncompliance, emotional distress and outbursts, and disruptive behavior, are common during the toddler and preschool years (Wakschlag et al., 2007). While many such behavior problems are developmentally normative, some young children exhibit clinically significant behavior problems that disrupt child and family functioning (Keenan & Wakschlag, 2002). Population‐based studies have reported prevalence estimates of elevated behavioral problems ranging from 7% to about 20% during the preschool years (Bayer et al., 2012; Briggs‐gowan et al., 2001; Egger & Angold, 2006; Stülb et al., 2019). Importantly, clinically significant early behavior problems may be predictive of later psychopathology and adverse outcomes in adolescence and adulthood (Campbell, 1995; Eisenberg et al., 2009; Mathiesen & Sanson, 2000; Prior et al., 1992), underscoring the importance of early identification and intervention.

Reliable and valid measures are central to assessment and treatment. For preschoolers, the Child Behavior Checklist for Ages 1.5–5 (CBCL/1.5–5) is one of the most commonly used measures for screening for behavior problems in both clinical and research settings (Ivanova et al., 2010; Medeiros et al., 2017), as it shows good reliability and validity in studies globally (Ivanova et al., 2010; Konold et al., 2003; Rescorla et al., 2011). In their initial validation, Achenbach and Rescorla (2001) described a factor structure of seven syndrome scales, which were then grouped under two second‐order factors of *Internalizing* and *Externalizing Problems*. Subsequent confirmatory factor analyses have generally found this structure holds in samples with different compositions of nationality, culture, sex, and diagnosis (Ivanova et al., 2010; Konold et al., 2003; Koot et al., 1997; Medeiros et al., 2017; Tan et al., 2007). Accumulating research evidence supports the use of the two broad domains of *Internalizing* and *Externalizing* as transdiagnostic constructs for profiling clinically significant behavior problems in young children (Achenbach et al., 2016; Krueger & Markon, 2011).

Despite basic psychometric validations of the CBCL/1.5–5 (e.g., reliability, validity, structure), questions remain about whether it measures behavior problems equivalently across samples with different demographic compositions (i.e., does the same score reflect similar levels of problems regardless of child or caregiver demographic characteristics?). This warrants further investigation, as previous research has shown that measured/observed levels of child behavior problems can be affected by child factors, including age, sex, bilingual status, and developmental level (Carneiro et al., 2016; Chen, 2010; Sun et al., 2021; Wakschlag et al., 2017), as well as by caregiver characteristics, including informant's primary language, sex (father vs. mother), socioeconomic status (SES), and mental health (Davis & Qi, 2022; Flouri et al., 2017; Müller et al., 2011; Schroeder et al., 2010). Given that observed raw score differences across demographic groups could result from measurement bias and/or *true* group differences, it is necessary to ensure measurement equivalence or account for measurement bias if they exist before concluding that measured differences across groups reflect actual differences in levels of behavior problems (Vandenberg & Lance, 2000). For example, developmentally, younger children might use *whining* (Item 97) to communicate more often regardless of their levels of internalizing problems, in which case, high scores on this item might not be as reflective of the *true* levels of internalizing problems in young children as in older children.

Analyses of measurement invariance (MI) and differential item functioning (DIF) allow examination of measurement bias across groups to facilitate the estimation of actual group differences after accounting for measurement bias. Surprisingly, despite its widespread use, little MI/DIF work has investigated whether the CBCL/1.5–5 functions equivalently across groups that differ by child and caregiver characteristics. We only identified three studies that investigated MI/DIF of the CBCL/1.5–5 (Dovgan et al., 2019; Gross et al., 2006; Rescorla et al., 2019). Rescorla and colleagues reported that the CBCL/1.5–5 Autism Spectrum Subscale functioned equivalently across ages 18 months, 3 years, and 5 years, demonstrating its potential utility for tracking longitudinal changes across the preschool years. In a study of children with autism spectrum disorder (ASD), Dovgan et al. (2019) found that CBCL/1.5–5 syndrome subscales, emotional reactivity, anxious/depressed, and somatic complaints were non‐invariant between those with and without intellectual disability (ID), raising concerns about the application of these syndrome scales in those with ASD and ID. When examining MI of *Internalizing* and *Externalizing* scales across groups defined by parent race/ethnicity, family income, and language version (English vs. Spanish), Gross et al. (2006) found differential functioning of items on both the *Internalizing* and *Externalizing* scales. However, their findings are limited by the selective set of grouping variables tested. In sum, studies of CBCL/1.5–5 support partial invariance of the scale across certain subgroups and further investigation is warranted to inform the use of CBCL/1.5–5 in diverse samples.

To date, no study has systematically assessed the MI/DIF of CBCL/1.5–5 across a broad range of child and caregiver‐level characteristics, likely due to the lack of a large enough sample with sufficient diversity across multiple relevant characteristics. This type of MI/DIF analysis is necessary to ensure that the measure can be reliably and validly employed to measure behavior problems in diverse groups of young children. Data from the National Institutes of Health (NIH) Environmental influences on Child Health Outcomes (ECHO) program provide a unique opportunity for a comprehensive psychometric examination of CBCL/1.5–5 across multiple child and caregiver characteristics. Therefore, the current study leveraged these data to examine the configural invariance and item‐level MI/DIF of CBCL/1.5–5 across a wide array of child and caregiver (i.e., respondent who completed the CBCL/1.5–5) characteristics that are available in the ECHO dataset and have been reported to influence caregiver‐report of child behavior problems. Moreover, we also explore MI/DIF across child race groups in the context of SES variables as sensitivity analyses.

## METHODS

### Participants

Data for the current study were drawn from the NIH ECHO program. For more information on ECHO, see Blaisdell et al. (2021). The aggregated ECHO dataset was queried to identify cases from 26 out of 69 cohorts that met the inclusion criteria: (a) CBCL/1.5–5 was administered to caregivers of children aged 18–71 months; (b) CBCL/1.5–5 item‐level data were available; (c) data were available on at least one of the child or caregiver‐characteristic variables of interest. When multiple timepoints of CBCL/1.5–5 data were available for a single child, the first administration was taken, yielding a final sample size of 9087 CBCL/1.5–5 administrations. See Table 1 for distributions of child and caregiver characteristics. For each MI/DIF testing, only individuals with data on the assessed variable(s) were included in the corresponding analyses (i.e., individuals missing data on any of the specific variables were excluded for the specific set of analyses), allowing for the largest inclusion sample possible for each analysis (see Table 1 for proportions of missing data by child and caregiver characteristics).

**TABLE 1:** Demographic information of the analytical sample of caregiver‐child dyads in Environmental influences on Child Health Outcomes (ECHO) (*N* = 9087).

Child Characteristics	
Female, *n* (%)	4327 (47.6%)
Age in months: M (SD)	39.2 (14.5)
Age subgroups, *n* (%)	
[18–27 m)	2784 (30.6%)
[27–36 m)	1585 (17.4%)
[36–45 m)	1408 (15.5%)
[45–54 m)	1259 (13.9%)
[54–72 m)	2051 (22.6%)
Race, *n* (%)	
White	4499 (49.5%)
Asian	494 (5.4%)
Black	1768 (19.5%)
Multiple race	1075 (11.8%)
Other	
American Indian or Alaska native^a^	146 (1.6%)
Native Hawaiian or other Pacific Islander^a^	42 (0.5%)
Other ‐participant self‐report	691 (7.6%)
Missing	372 (4.1%)
Ethnicity, *n* (%)	
Hispanic	2923 (32.2%)
non‐Hispanic	6019 (66.2%)
Missing	145 (1.6%)
Bilingual home environment, *n* (%)	
Yes	2931 (32.3%)
No	5056 (55.6%)
Missing	1100 (12.1%)
Any NDD, *n* (%)	
Yes	713 (7.8%)
No	4888 (53.8%)
Missing	3486 (38.4%)
Intellectual disability^b^	37 (0.4%)
Developmental delay^b^	225 (2.5%)
Autism, ASD, or Pervasive Developmental Disorder^b^	112 (1.2%)
Attention Deficit Disorder or Attention Deficit/Hyperactivity Disorder^b^	46 (0.5%)
Speech disorder^b^	568 (6.3%)
Learning disability^b^	84 (0.9%)
Respondent characteristics	
Female respondent, *n* (%)	8726 (96%)
Biological mother respondent, *n* (%)	8496 (93.5%)
CBCL language of administration, *n* (%)	
English	7596 (83.6%)
Spanish	923 (10.2%)
Missing	568 (6.3%)
Highest caregiver education, *n* (%)	
Less than high school	731 (8%)
High school degree or equivalent	1789 (19.8%)
Some college	2213 (24.4%)
Bachelor's degree	2148 (23.6%)
Master's degree or higher	2001 (22%)
Missing	196 (2.2%)
Household income, *n* (%)	
Less than $30,000	2084 (22.9%)
$30,000‐$49,999	847 (9.3%)
$50,000‐$74,999	1028 (11.3%)
$75,000‐$99,999	748 (8.2%)
$100,000 or more	2837 (31.2%)
Missing	1543 (17%)
Caregiver dep. T score (M, SD)	46.5 (8.5)
Caregiver depression, *n* (%)	
*T* < 60	4709 (51.8%)
*T* ≥ 60	333 (3.7%)
Missing	4045 (44.5%)

^a^ For the MI/DIF analysis, categories of “American Indian or Alaska Native” and “Native Hawaiian or other Pacific Islander” were collapsed into “Other”.

^b^ Denominators used for calculating percentages of children with each NDD include missing values.

### Measures

**CBCL/1.5–5** (Achenbach & Rescorla, 2001) requires caregivers to rate their child's behaviors “now or within the past 2 months” on items describing behavior problems on a three‐point scale: 0 “Not True”, 1 “Somewhat or Sometimes True”, to 2 “Very True or Often True”. The ***Internalizing*** scale includes 36 items (score range: 0–72) across syndrome scales of Emotionally Reactive, Anxious/Depressed, Somatic Complaints, and Withdrawn; the ***Externalizing*** scale includes 24 items (score range: 0–48) across syndrome scales of Attention Problems and Aggressive Behaviors; and the ***Total Problems*** include all items from both the *Internalizing* and *Externalizing* problems, with additional items from syndrome scales of Sleep Problems and Other Problems, totaling 99 items (score range: 0–198). Frequencies of item endorsement are provided in Table S1. *Internalizing, Externalizing,* and *Total Problems* are the constructs of primary interest in the current analyses.

### Grouping variables for measurement invariance/differential item functioning testing

We tested MI/DIF across univariate groupings defined by single child and caregiver characteristics separately, and across multivariate groupings defined by two child and caregiver variables in combination. Throughout, we use the term *grouping* to refer to subgroups defined by various levels of a given characteristic or combination of characteristics.

#### Univariate groupings

*Child characteristics* of interest included biological sex (male vs. female), age at the administration of CBCL/1.5–5 (18–27, 27–36, 36–45, 45–54, and 54–72 months, with children at the group boundaries assigned to the older group), bilingual/multilingual status (yes vs. no), and caregiver‐reported diagnosis of a neurodevelopmental disorder (NDD) (none vs. any). For child bilingual and NDD variables, we included data from the same timepoint (i.e., within 6 months of CBCL/1.5–5 administration date) when available and otherwise used historic data from the closest previous timepoint to keep more cases for analysis. Bilingual status was determined using a harmonized indicator of bilingual/multilingual exposures, incorporating direct reports of bilingual exposure and language of administration for child tests within ECHO. We used the following NDD diagnoses reported on the ECHO medical history form: ASD, intellectual/developmental disorders, attention deficit/hyperactivity disorder, learning disability, and speech disorder. If the child was reported to have one or more of these diagnoses, they were classified as having NDD.

*Caregiver characteristics* included respondent sex (male vs. female), language version of CBCL administration (English vs. Spanish; the only two available in the ECHO data), annual household income levels (<$30,000; $30,000‐$49,999; $50,000‐$74,999; $75,000‐$99,999; ≥$100,000), caregiver education level, and caregiver depression. Given the low number of male caregivers completing CBCL/1.5–5, caregiver educational level and depression status were only examined for female caregivers (mostly mothers). Education level was harmonized to derive four categories: less than high school; high school diploma or equivalent; some college; Bachelor's degree and above. Caregiver depression was indexed using the PROMIS® Depression T score (mean of 50 and standard deviation of 10 normed in reference to U.S. adults) as a common metric to which multiple instruments have been linked (Blackwell et al., 2021; Choi et al., 2014; Kaat et al., 2017). For the current analyses, caregiver depression was dichotomized to form groups according to the recommended threshold *T* score = 60 to distinguish cases with elevated depression levels. For caregiver depression status, only data from the same timepoint of CBCL/1.5–5 were used; for other caregiver demographic variables, historic data from the closest previous timepoint was used when the variable was not available from the same timepoint as CBCL/1.5–5.

#### Multivariate groupings

To understand the differential impact of variables on MI/DIF, we examined multivariate groupings defined by combinations of child or caregiver characteristics. Based on data availability and known confounding impacts on child behavior problems, we assessed MI/DIF across groupings defined by: (a) child sex and age; (b) child sex and NDD diagnosis (May et al., 2019); and (c) caregiver education and income levels (Braveman et al., 2005). To yield sufficient sample sizes, caregiver education was collapsed into two categories (less than Bachelor's degree, Bachelor's degree or higher) and income was collapsed into three categories (<$50,000, $50,000‐$74,999, $75,000 or more).

*Sensitivity Analyses.* We conceptualized race as a social‐cultural variable that is confounded with SES variables (Cheng, Goodman, & The Committee on Pediatric Research, 2015) and thus, conducted sensitivity analyses to evaluate the unique impact of child race/ethnicity on MI/DIF, as previous studies have shown the impact of race on the measurement of behavior problems and psychopathology in children (Gross et al., 2006; Vaughn‐Coaxum et al., 2016). Race categories were collapsed to yield sufficient sample sizes: White, Black, Asian, Multiple Race, and Other (see Table 1 for all available racial groups). Ethnicity categories were not examined, given insufficient sample sizes. We chose to focus on child race, rather than the respondent race, to capture the full picture of the child's developmental context as it reflects the race of both parents. We tested combinations of race and caregiver education level, as well as race and income level (collapsed into two categories: <$50,000, $50,000 or more), to disentangle the impact of race in the context of SES variables.

Given language version variable is potentially confounded with other variables (i.e., bilingual status, race/ethnicity, caregiver education level, household income level), we conducted DIF testing across multivariate groupings between language version and bilingual status and caregiver education level where the sample sizes were sufficient for analyses (i.e., more than 100 cases within each level of combined groupings).

### Statistical analyses

Separate analyses were conducted for each of the univariate and multivariate groupings for each latent construct of *Internalizing, Externalizing,* and *Total Problems*. All item response theory (IRT) models were estimated using the graded response model (Samejima, 1997) using the *mirt* package (Chalmers, 2012) in R 4.1.0 (R Core Team, 2022).

#### Configural invariance testing

Establishing configural invariance (similar number of factors and loading pattern) is the first step in MI/DIF examination. For *Internalizing* and *Externalizing*, we estimated a unidimensional model in each subsample. For *Total Problems*, we estimated both a unidimensional model and a bifactor model (i.e., *Internalizing* and *Externalizing* items loaded on two separate factors and all items loaded on the general factor representing *Total Problems*, with all factors mutually orthogonal). Model fit was evaluated using the standardized root mean squared residual (SRMSR) and root mean squared error of approximation (RMSEA), given their demonstrated performance advantage in IRT models (Maydeu‐Olivares & Joe, 2014) and the general incomparability of other estimable fit measures (e.g., comparative fit index [CFI], Tucker‐Lewis index [TLI]) from IRT to commonly used criteria (Yuan & Chan, 2005). Standardized root mean squared residual below 0.08 was used as the criterion for adequate fit (Hu & Bentler, 1999), while RMSEA is reported as a secondary index of fit. For *Total Problems*, the unidimensional and bifactor models were compared with respect to Akaike information criterion (AIC) and sample size‐adjusted Bayesian Information Criterion (SABIC), where a difference greater than 10 indicated a superior fit for the model with lower deviance (Raftery, 1995).

#### Alignment method for measurement invariance/differential item functioning estimation

We applied the alignment method for MI/DIF testing in the current study to accommodate the considerable number of items included in the analyses and the need to examine DIF across univariate and multivariate groupings. In brief, alignment involves estimating the configural model separately in each group and then estimating factor means and variances across groups within each grouping such that DIF is minimized for that grouping. Alignment yields DIF‐adjusted factor mean and variance estimates, as well as “aligned” item parameter estimates and standard errors, which can be used to test for DIF and judge its impact on item responses. We refer interested readers to Appendix S1, Mansolf et al. (2020), and Muthén and Asparouhov (2014) for technical details of the alignment method.

#### Statistical testing of measurement invariance/differential item functioning

We tested DIF for statistical significance using ANOVA, treating parameter estimates and standard errors analogously to sample statistics (mean and standard error, respectively) in conventional ANOVA, to screen for significance to examine the impact of DIF. For each grouping, an item was determined to have statistically significant DIF if any Bonferroni‐corrected *p* value was less than 0.05, where the correction was applied for all item parameters. For multivariate groupings, two‐way ANOVA was used, allowing DIF by each variable and the interaction between variables to be partitioned. To evaluate the differential impact of variables on MI/DIF, η^2^ statistics were calculated for the main and interaction effect of DIF on each item, allowing comparisons of impact on the magnitude of DIF among the three sources. See Appendix S1 for additional details.

#### Assessing the impact of measurement invariance/differential item functioning

To steer away from reliance on significance testing, we used the unsigned item difference in the sample (UIDS; Meade, 2010) to quantify the impact of DIF on CBCL items and construct scores (i.e., *Internalizing, Externalizing, and Total Problems*). Unsigned Item Difference in the Sample is calculated by comparing expected item scores using model parameters estimated from different groups, and the magnitude of UIDS reflects the impact of differences in item parameter estimates on scores and can be interpreted on the scale of raw item scores. Using a threshold of UIDS >0.1, representing one‐tenth of a point of score difference on the item, we identified items with the most impactful DIF for each construct, operationalized as those with UIDS >0.1 across more than one univariate grouping for *Internalizing* and *Externalizing*, and more than two for *Total Problems*. Thus, removing these items yielded a more measurement‐invariant (i.e., robust) item set for each construct. Moreover, to assess the aggregated impact of item‐level DIF, Signed Test Difference in the Sample (STDS) was calculated to represent differential test functioning. See Appendix S1 for additional details on UIDS and STDS calculation.

The effect of removing these items on reliability was assessed by plotting IRT reliability of measurement, calculated as one minus the reciprocal of test information, as a function of the latent trait, comparing values from the full item set to the robust item set.

#### Linking of robust and full item sets

For each domain, we used equipercentile equating (Kolen & Brennan, 2004), implemented in the *equate* package in R (Albano, 2016) to derive crosswalk tables which can be used to link total scores from the robust item sets to the total score metric of the original full item set. Then, the linked total scores can be used to derive the corresponding T scores using conversion tables available from the CBCL scoring manual. Correlations and mean differences between the scores from the full (i.e., originally published scale) and the linked scores from the robust item sets were computed to assess the reliability and bias, respectively, of the resulting linkages. Lastly, we conducted group comparisons of T scores based on the robust item sets across levels of each child and caregiver characteristic to examine whether substantive group differences remain after removing items with impactful measurement bias.

## RESULTS

### Configural invariance of CBCL/1.5–5

**FIGURE 1:**
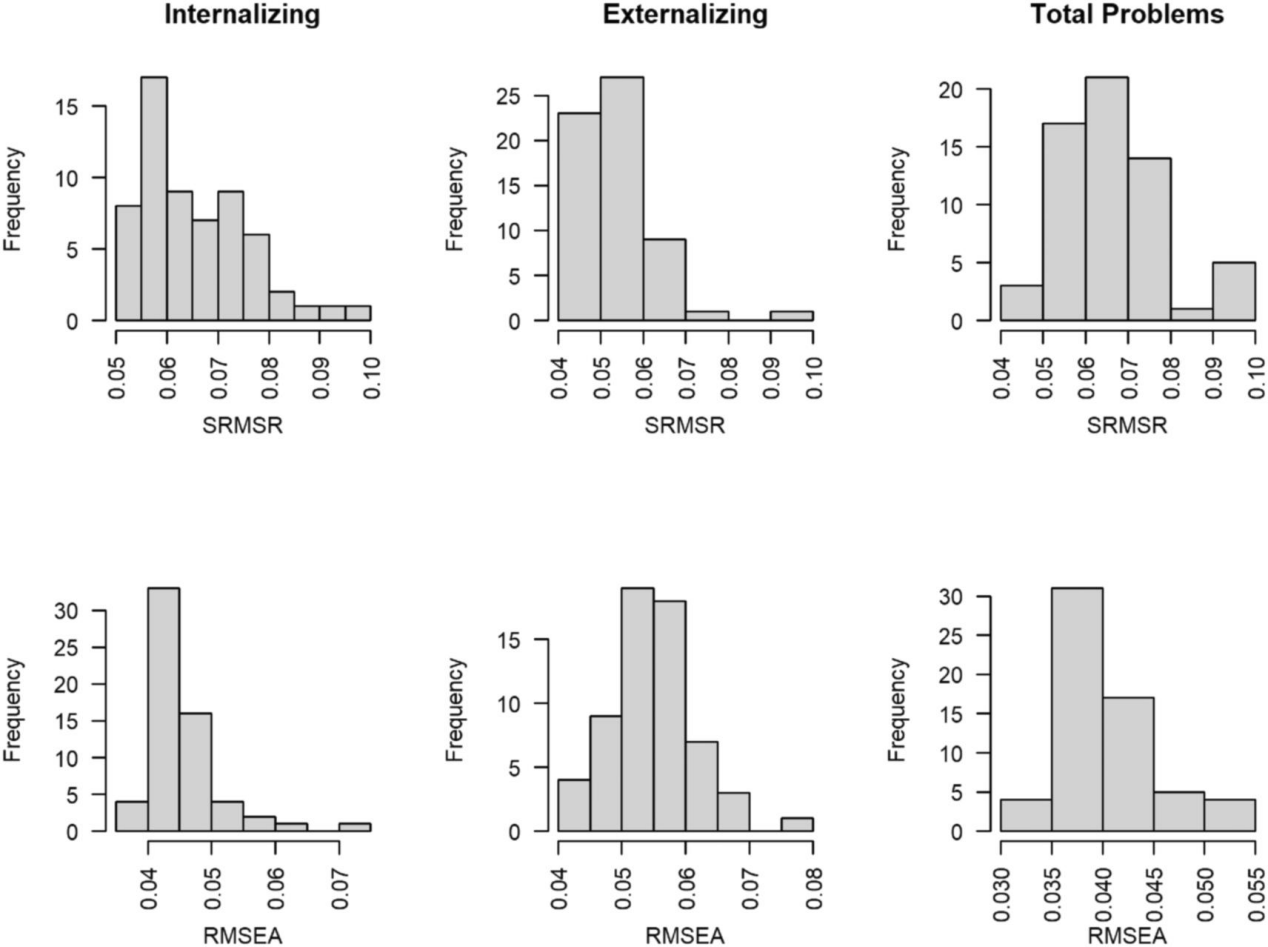
Model fit distributions for factor analysis models across three latent constructs. SRMSR, Standardized Root Mean Squared Residual; RMSEA, Root Mean Squared Error of Approximation.

Both AIC and SABIC indicated that the bifactor model fit better than the unidimensional model across all subsamples for *Total Problems*. We, therefore, used the bifactor model parameters for DIF testing. Configural invariance was tested across all groupings of interest. Figure 1 shows the distribution of SRMSR and RMSEA values across groupings for each latent construct, estimated using the unidimensional model for *Internalizing* and *Externalizing* and the bifactor model for *Total Problems*. While most models across groupings and constructs showed adequate fit, model fit was better on average for *Externalizing* (mean SRMSR = 0.054) than for *Internalizing* (0.065) or *Total Problems* (0.066). Full sets of fit statistics and deviance can be found in the Open Sicence Framework (OSF) Repository: https://tinyurl.com/CBCLosffiles, due to space limit. Syndrome Scale analyses results are presented in the Appendix S2 and Supplementary Tables S7 to S11 and Figures S3, S4, and S6 to S11 for interested readers.

### Significance and meaningfulness of item‐level differential item functioning

Given the large number of results generated and the limited manuscript space, below we focus on the main takeaways of our findings, summarizing alignment results with respect to specific assessments of DIF in the CBCL 1.5–5. Detailed results of item parameter estimates, significance, effect sizes (η^2^) of DIF, and UIDS statistics across all groupings and constructs can be found in the OSF repository.

Across the child and caregiver groupings we tested, we observed significant DIF in most of the items across groupings. Only a small set of items showed no DIF across child characteristics tested (see Table 2), while all items showed DIF across caregiver characteristics tested. Because this number of items was so small and not representative of the breadth of the full CBCL 1.5/5, we instead sought to identify which items had the *most* DIF and, when removed, would yield a robust item set with scores comparable across diverse samples.

**TABLE 2:** Items without any significant differential item functioning (DIF) across domains and characteristics.

Latent construct	Characteristics	Item	CBCL subdomain
Externalizing	Child	27 lacks guilt	Aggressive behavior
		53 attacks people	Aggressive behavior
Internalizing	Child	07 Can't stand things out of place	Somatic complaints
		70 little affection	Withdrawn
		86 too concerned with neatness	Somatic complaints
		51 panics	Emotionally reactive
		62 refuses active games	Withdrawn
		93 vomits	Somatic complaints
		98 withdrawn	Withdrawn
Total problems	Child	41 holds breath	Other problems
		62 refuses active games	Withdrawn
		93 vomits	Somatic complaints

**FIGURE 2:**
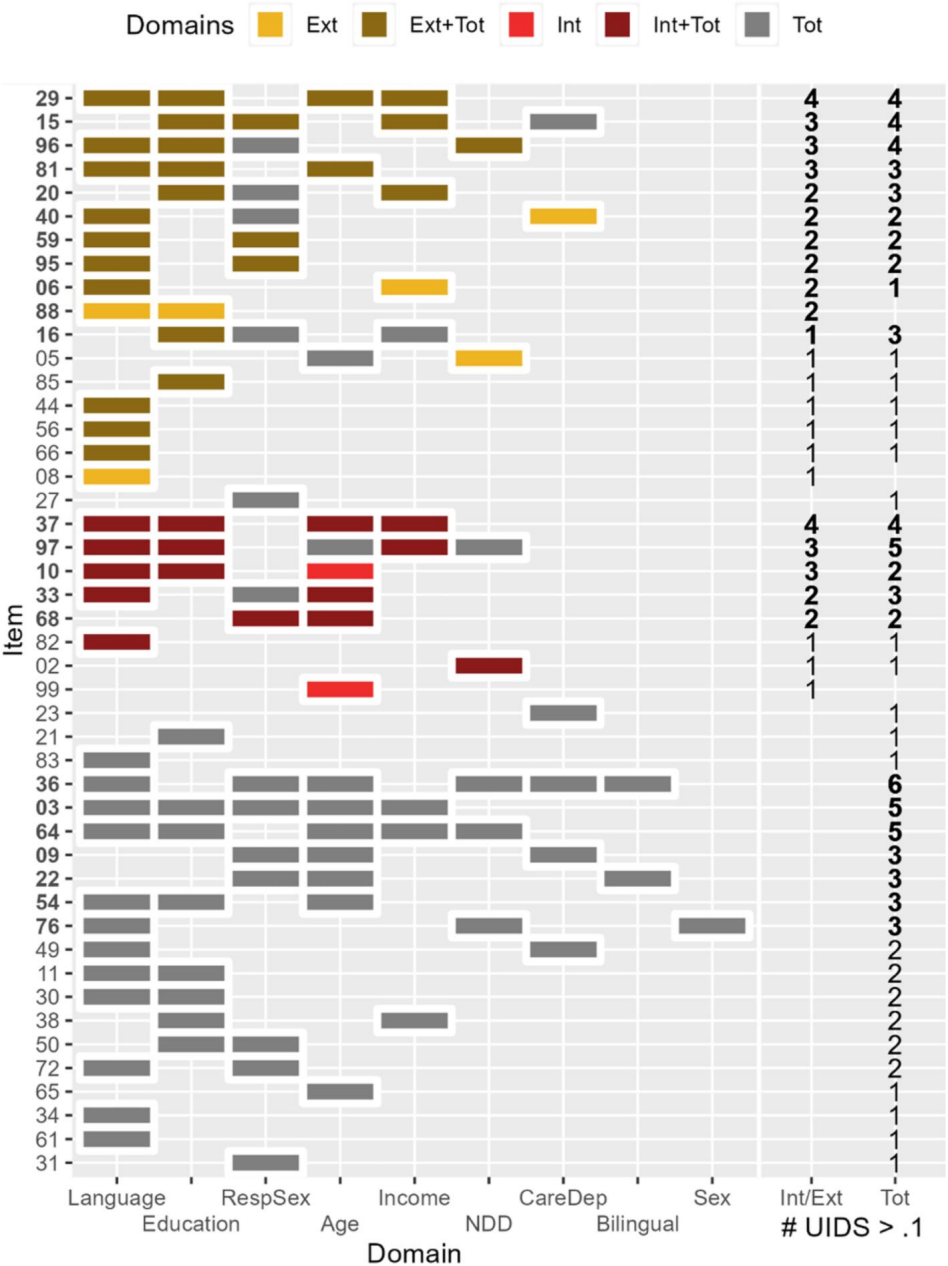
Items with Unsigned Item Difference in the Sample (UIDS) > 0.1 in Univariate Groupings across Latent Constructs. *Ext*, Externalizing domain; *Ext + Tot*, Externlizing and Total Problems; *Int*, Internalizing domain; *Int + Tot*, Internalizing and Total Problems; *Tot*, Total Problems; *UIDS*, Unsigned Item Difference in the Sample; *RespSex*, Respondent Sex; *NDD*, Neurodevelopmental Disorder. Items with no significant differential item functioning (DIF) or no UIDS >0.1 are excluded from the figure. Items are grouped by domains and sorted in decreasing order of the number of groupings with UIDS >0.1 of items; groupings are sorted by the number of items with UIDS >0.1 within the grouping. The numbers on the two right‐hand columns show the number of groupings with UIDS >0.1 for the specific items within domains (Internalizing or Externalizing, and Total Problems).

To better understand the meaningfulness of DIF in the CBCL 1.5/5, Figure 2 presents items and groupings in which DIF was found to be significant and with UIDS greater than 0.1. For *Internalizing,* most of the impactful DIF (i.e., UIDS >0.1) was aggregated on items 10 *too dependent,* 33 *feelings hurt*, 37 *upset by separation*, 68 *self‐conscious*, and 97 *whining*. For *Externalizing*, impactful DIF was observed on items 06 *can't sit still*, 15 *defiant*, 20 *Disobedient*, 29 *frustrated*, 40 *Hits Others*, 59 *quickly shifts*, 81 *stubborn,* 88 *uncooperative,* 95 *wanders away*, and 96 *wants attention*. For *Total Problems*, impactful DIF was observed across 16 items (03, 09, 15, 16, 20, 22, 29, 33, 36, 37, 54, 64, 76, 81, 96, 97) showing UIDS >0.1 on at least three out of nine univariate groupings tested. Taken together, the impact of DIF was higher for *Externalizing* than *Internalizing* and was the highest for *Total Problems*. Distributions of median UIDS of all items can be found in Figure S1.

**FIGURE 3:**
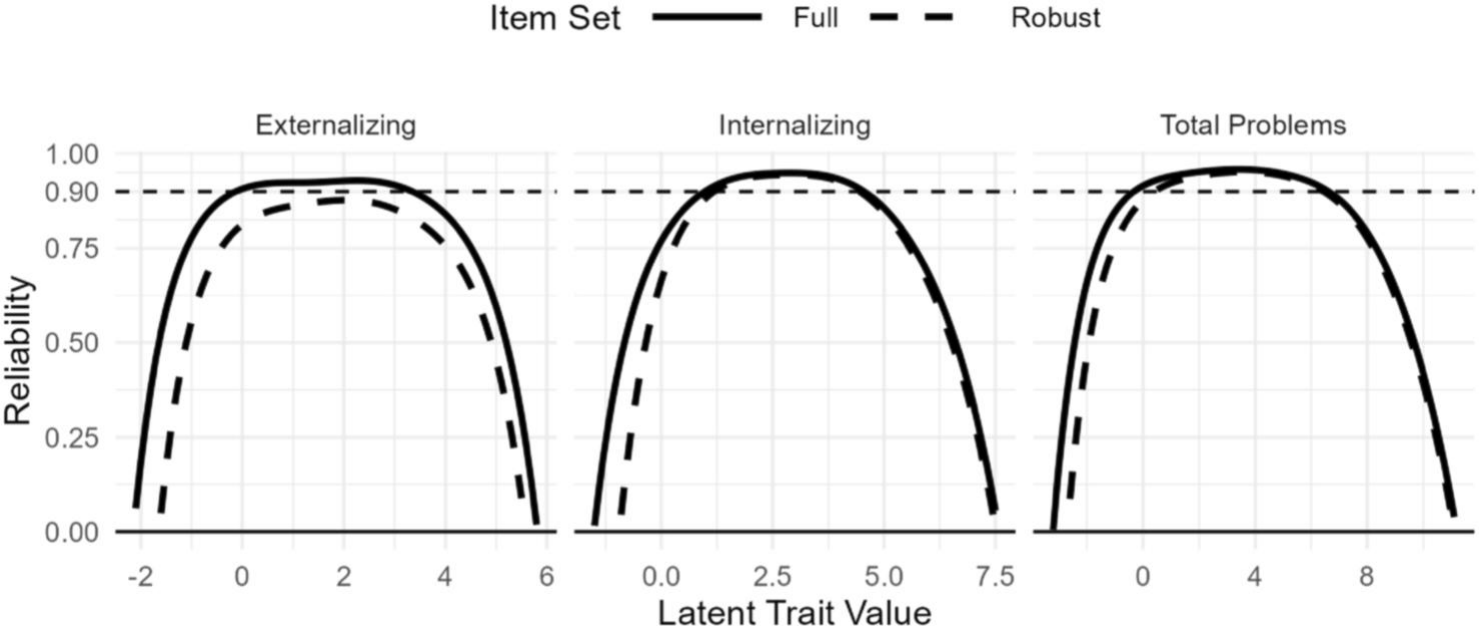
Item response theory (IRT) Reliability for Full and Robust Item Sets. *Full*, full CBCL item set; *Robust*, item set with high‐DIF items removed.

Table 3 contains the robust item sets for each construct (i.e., with high DIF items removed): *Internalizing* (*n*_item_ = 31), *Externalizing* (*n*_item_ = 14), and *Total Problems* (*n*_item_ = 83). Reliabilities for the full and the robust items were all near or above 0.9 and in nearly identical ranges for Internalizing and Total Problems (see Figure 3 with the solid and dotted lines closely overlapping).

**TABLE 3:** CBCL/1.5‐5 robust item sets with less differential item functioning for internalizing, externalizing, and total problems.

Internalizing	Externalizing	Total problems
01, 02, 04, 07, 12, 19, 21, 23, 24, 39, 43, 45, 46, 47, 51, 52, 62, 67, 70, 71, 78, 79, 82, 83, 86, 87, 90, 92, 93, 98, 99	05, 08, 16, 18, 27, 35, 42, 44, 53, 56, 58, 66, 69, 85	01, 02, 04, 05, 06, 07, 08, 10, 11, 12, 13, 14, 17, 18, 19, 21, 23, 24, 25, 26, 27, 28, 30, 31, 32, 34, 35, 38, 39, 40, 41, 42, 43, 44, 45, 46, 47, 48, 49, 50, 51, 52, 53, 55, 56, 57, 58, 59, 60, 61, 62, 63, 65, 66, 67, 68, 69, 70, 71, 72, 73, 74, 75, 77, 78, 79, 80, 82, 83, 84, 85, 86, 87, 88, 89, 90, 91, 92, 93, 94, 95, 98, 99

**FIGURE 4:**
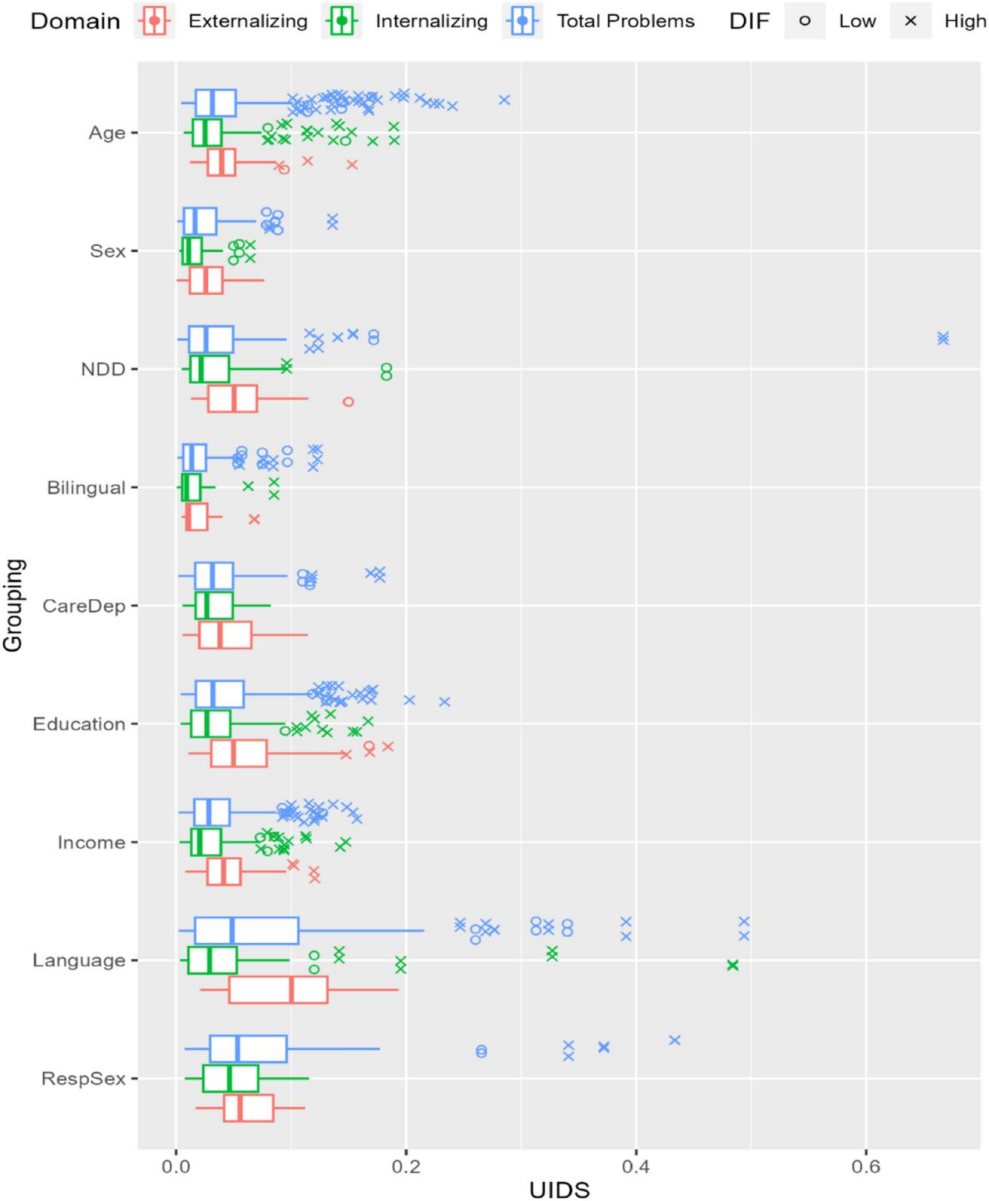
Distribution of Unsigned Item Difference in the Sample (UIDS) by Grouping and Latent Constructs. *NDD*, Any Neurodevelopment Disorders; *CareDep*, Caregiver Depression with a cutoff of T‐score ≥ 60 for clinical range; *RespSex*, Respondent Sex.

**FIGURE 5:**
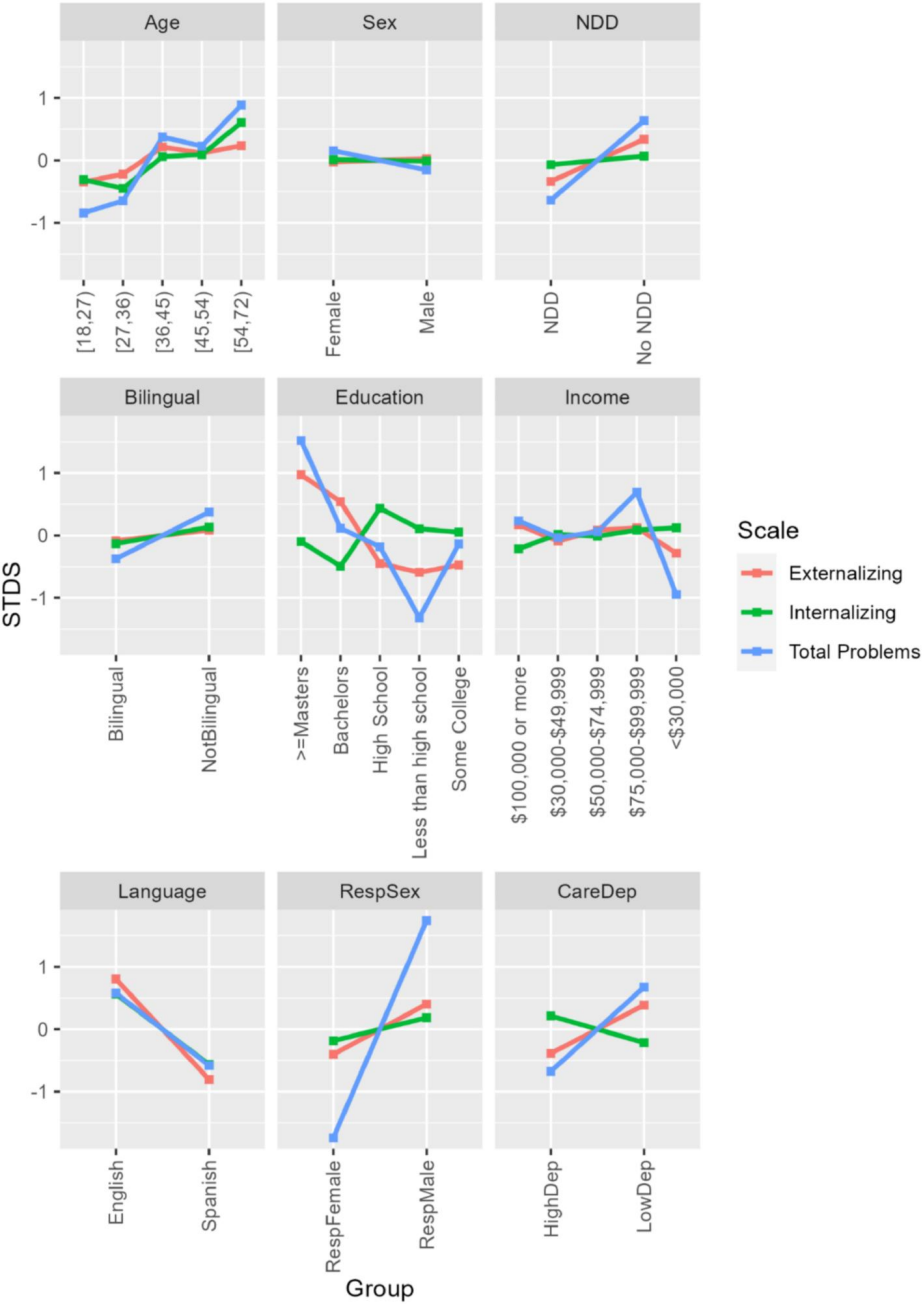
Signed Test Difference in the Sample (STDS) for Univariate Groupings. *RespSex*, Respondent Sex; *RespFemale*, Female Respondent; *RespMale*, Male Respondent; *CareDep*, Caregiver Depression with clinical threshold of *T* = 60; *HighDep*, Depression *T* score greater than or equal to 60; *LowDep*, Depression *T* score less than 60; *NDD*, Neurodevelopmental Disorder.

To understand the impact of different groupings, Figure 4 presents the distribution of UIDS by univariate groupings and domains for items with significant DIF. Notably, the impactful DIF across items concentrated on language version, caregiver education level, caregiver sex, and child age. The largest sources of DIF for caregiver characteristics were language version of CBCL administration, followed by caregiver sex and education. The largest source of DIF for child characteristics was child age. Effect sizes of the multivariate groupings on DIF (Table 4) showed main effects of the groupings were associated with larger magnitudes of DIF than the interactions. Notably, child age and NDD status were associated with larger DIF than child sex or their interactions with child sex. One outlier item (Item 76, *speech problem*) showed the largest magnitudes of DIF between those with and without NDD. Household income level showed larger magnitudes of DIF effect sizes than caregiver education level, especially for *Externalizing Problems*. For differential test functioning, respondent sex showed the largest STDS on the measurement of *Total Problems*, followed by caregiver education levels on *Total Problems* (see Figure 5).

**TABLE 4:** Median and maximum effect sizes (eta‐squared) of main and interaction effects on differential item functioning (DIF).

	Term	Internalizing		Externalizing		Total problems	
		Median	Max	Median	Max	Median	Max
Caregiver education‐income	Education	0.091	0.672	0.072	0.680	0.093	0.648
	Income	0.164	0.817	0.267	0.699	0.208	0.845
	Education: Income	0.140	0.623	0.089	0.517	0.140	0.629
Child sex‐age	Age	0.286	0.891	0.334	0.858	0.377	0.970
	Sex	0.035	0.660	0.037	0.489	0.035	0.736
	Sex: Age	0.130	0.397	0.126	0.436	0.138	0.421
Child Sex‐NDD	Sex	0.073	0.648	0.108	0.557	0.092	0.784
	NDD	0.261	0.902	0.145	0.864	0.175	0.979
	Sex: NDD	0.053	0.317	0.062	0.546	0.061	0.552

Sensitivity analyses with child race groupings showed impactful DIF across race categories on multiple items on each latent construct, with impactful DIF aggregated on similar items as identified above. While child race, caregiver education, and income all showed unique associations with DIF, child race was associated with larger magnitudes of DIF than the SES variables after adjusting for each other and the interaction, indicating that measurement bias across racial groups cannot be fully accounted for by SES variables (See Table S2). Similarly, the multivariate analyses with language version revealed that language version was associated with more DIF than child bilingual status but with less DIF than caregiver education level (See Table S3 for effect sizes, Figure S2 for Model Fit, and Table S6 for items without significant DIF and Figure S5 for items with impactful DIF).

### T scores based on the robust item sets and tests of group differences

Correlations between linked scores (i.e., linking summed scores for the robust item sets to the metric of the full CBCL) and the observed summed scores of the full item sets, were 0.963 for *Internalizing*, 0.965 for *Externalizing*, and 0.989 for *Total Problems*. The raw mean differences between linked and observed scores, an indicator of linking bias, were −0.004, 0.008, and 0.007 for *Total Problems*, *Internalizing*, and *Externalizing*, respectively, indicating very minimal bias (See Table S4). Group means and standard deviations of T scores based on robust item sets, and ANOVA tests of mean differences in each grouping, are presented in Table 5 (group comparisons of the full item set are shown in Table S5). We observed the largest group differences in T scores based on robust item sets across Caregiver Education levels (*η* ^2^ ≥ 0.023) on all three latent constructs, followed by income (*η* ^2^ ≥ 0.020) and Caregiver Depression (*η* ^2^ ≥ 0.018), with the smallest differences in *Internalizing* and the largest differences in *Total Problems*. For child characteristics, Child NDD status (*η* ^2^ ≥ 0.021) showed small to medium effects, with the smallest differences in *Externalizing.*

**TABLE 5:** Group comparisons of T‐scores of robust item set across child and caregiver characteristics across domains.

Grouping variable	Group	N	Externalizing	Internalizing	Total problems
Child characteristics					
Age (months)			F(4,7380) = 21.918; p = 0.000, η^2^ = .012	F(4,7380) = 18.866; p = 0.000, η^2^ = .010	F(4,7380) = 21.329; p = 0.000, η^2^ = .011
	[18,27)	2241	45.18 (9.54)	42.65 (9.55)	44.00 (9.57)
	[27,36)	1328	46.46 (10.15)	45.20 (10.36)	46.28 (10.26)
	[36,45)	1155	45.96 (10.58)	45.16 (10.49)	45.68 (10.77)
	[45,54)	983	44.14 (10.30)	43.88 (10.15)	43.69 (10.13)
	[54,72)	1678	43.43 (10.07)	44.36 (10.45)	43.48 (10.31)
Child sex			F(1,7381) = 41.844; p = 0.000, η^2^ = .006	F(1,7381) = 12.967; p = 0.000, η^2^ = .002	F(1,7381) = 29.021; p = 0.000, η^2^ = .004
	Female	3527	44.21 (9.97)	43.61 (10.15)	43.85 (10.13)
	Male	3856	45.72 (10.16)	44.46 (10.20)	45.12 (10.21)
Bilingual			F(1,6507) = 1.951; p = 0.162, η^2^ < .001	F(1,6507) = 0.352; p = 0.553, η^2^ < 0.001	F(1,6507) = 0.003; p = 0.954, η^2^ < 0.001
	Bilingual	2345	44.75 (10.27)	44.02 (10.22)	44.45 (10.33)
	Not bilingual	4164	45.12 (10.06)	43.87 (10.18)	44.46 (10.15)
NDD			F(1,4491) = 95.068; p = 0.000, η^2^ = .021	F(1,4491) = 126.232; p = 0.000, η^2^ = .027	F(1,4491) = 138.176; p = 0.000, η^2^ = .030
	NDD	552	49.26 (12.42)	49.07 (12.26)	49.65 (12.48)
	No NDD	3941	44.76 (9.79)	43.91 (9.77)	44.25 (9.74)
Caregiver characteristics					
Caregiver depression			F(1,4038) = 136.745; p = 0.000, η^2^ = .033	F(1,4038) = 75.894; p = 0.000, η^2^ = .018	F(1,4038) = 143.201; p = 0.000, η^2^ = .034
	HighDep	247	52.75 (11.78)	49.85 (11.56)	52.49 (11.46)
	LowDep	3793	44.91 (10.09)	43.97 (10.19)	44.40 (10.21)
Income			F(4,6148) = 41.263; p = 0.000, η^2^ = .026	F(4,6148) = 30.816; p = 0.000, η^2^ = .020	F(4,6148) = 67.041; p = 0.000, η^2^ = .042
	<$30,000	1583	42.97 (9.16)	42.33 (9.14)	41.90 (8.90)
	$30,000‐$49,999	681	46.17 (10.67)	44.62 (10.68)	45.63 (10.77)
	$50,000‐$74,999	838	45.27 (9.73)	43.98 (9.86)	44.69 (9.81)
	$75,000‐$99,999	602	44.34 (9.60)	42.89 (9.68)	43.54 (9.50)
	$100,000 or more	2301	46.86 (11.00)	45.76 (11.06)	46.94 (11.18)
Language version			F(1,6956) = 18.961; p = 0.000, η^2^ = .003	F(1,6956) = 17.236; p = 0.000, η^2^ = .002	F(1,6956) = 47.130; p = 0.000, η^2^ = .007
	English	6193	44.68 (10.07)	43.79 (10.05)	44.08 (10.12)
	Spanish	765	46.36 (10.10)	45.40 (10.89)	46.75 (10.37)
Maternal education			F(4,7227) = 63.130; p = 0.000, η^2^ = .034	F(4,7227) = 42.248; p = 0.000, η^2^ = .023	F(4,7227) = 93.252; p = 0.000, η^2^ = .049
	<high school	545	43.92 (9.48)	43.12 (9.74)	43.11 (9.50)
	High school	1451	46.51 (10.44)	45.08 (10.57)	46.33 (10.61)
	Some college	1741	48.08 (11.19)	47.38 (11.46)	48.76 (11.50)
	Bachelor's degree	1762	42.45 (8.80)	42.07 (9.20)	41.53 (8.61)
	>=Master's degree	1676	46.38 (10.57)	44.95 (10.30)	45.98 (10.49)
Respondent sex			F(1,7383) = 2.865; p = 0.091, η^2^ < 0.001	F(1,7383) = 0.108; p = 0.743, η^2^ < 0.001	F(1,7383) = 1.342; p = 0.247, η^2^ < 0.001
	Female	7061	44.96 (10.15)	44.06 (10.24)	44.48 (10.25)
	Male	324	45.93 (8.85)	43.87 (8.88)	45.15 (8.79)

## DISCUSSION

The CBCL/1.5–5 has been widely applied in clinical practice and research, but little information is available on its measurement equivalence across subgroups defined by various child and caregiver characteristics. We conducted the largest study of MI/DIF of CBCL/1.5–5 (*N* = 9087) to date and identified multiple sources of measurement bias across child and caregiver characteristics. Our findings directly inform the use of CBCL/1.5–5 and have implications for the measurement of childhood behavior problems more broadly.

Results of the factor analyses confirmed the unidimensional structure of *Internalizing* and *Externalizing* broad domains and the bifactor structure of *Total Problems* across child and caregiver groupings, demonstrating that both symptom clusters and the general psychopathology (“*p*”) factor account for distinct sources of variance in ratings of child psychopathology. These findings are consistent with prior conceptualizations of the CBCL/1.5–5 (Achenbach & Rescorla, 2001) and previous studies in different samples (Achenbach et al., 2016), providing further evidence for the utility of broad‐domain constructs.

Item‐level analyses identified multiple sources of DIF, raising concerns about the ubiquitous application of CBCL/1.5‐5 in diverse samples without adjusting for measurement bias. Caregiver demographic variables were associated with a larger magnitude of measurement bias than child characteristics across all three constructs as indicated by UIDS and STDS, with the greatest DIF/Differential Test Functioning (DTF) arising from language version, caregiver education, and respondent sex. Among them, language version showed the largest impact of MI/DIF across all constructs. It is possible that both the information loss due to translation and differences in cultural expectations/interpretations of child behavior problems contribute to the noninvariance of the English and Spanish versions of the CBCL/1.5–5. For example, as discussed by Gross et al., 2006, Item 97 *whining* is translated to *queja* in the Spanish version, which means *complain* in English and may have different connotations for parents. This underscores the need to consider validation efforts separately from simple translation of measurement tools in order to ensure equivalence across language translations.

We further observed an impact of respondent sex and caregiver education on DIF. It is possible that parents' ethnotheories of desirable and maladaptive behaviors in children (Olson et al., 2019), and their expectations about child development, influence how parents perceive and rate their child's behavior. Hence, in line with recommendations to consider how caregiver informants' background might influence the assessment of child psychopathology (De Los Reyes & Kazdin, 2005), these findings provide empirical evidence of the potential impact of caregiver characteristics on the measurement of specific child behavior problems. Notably, we did not observe much impactful DIF related to caregiver depression, despite the depression‐distortion hypothesis suggesting that depressed caregivers perceive more problems in their children (De Los Reyes & Kazdin, 2005). Since only a small proportion of caregivers met the clinical cutoff of T‐score ≥60 (3.4%), our analyses might have lacked the power to detect possible bias across caregiver depression status. Nevertheless, we found small‐to‐moderate differences in *Internalizing, Externalizing, and Total Problems* across caregiver depression groups using T‐scores derived from robust item sets, indicating that observed differences across caregiver depression groups were not attributable to item bias (Chi & Hinshaw, 2002; Gartstein et al., 2009).

Group comparison of T scores using robust item sets showed that differences between groups of language version and respondent sex were close to zero, while the original scores of *Internalizing* differed significantly across language version and respondent sex (see Table S5). These findings further underscore the need to assess and account for measurement bias resulting from informant characteristics when interpreting results from caregiver‐report measures. For example, father‐mother discrepancies in perceived levels of child behavior problems have been reported by many previous studies but were not significant in our sample after accounting for measurement bias. This is likely because the observed discrepancies are at least partially due to differences in how caregivers report specific items. Thus, while DIF is not always problematic and, in fact, often informative for measure development and application by showing group‐specific patterns of item responding, accounting for DIF across these factors is necessary for teasing apart measurement bias from true differences in the construct of interests (e.g., behavior problems) between groups.

Child age showed a larger impact on DIF than child sex, indicating the importance of developmental considerations when measuring psychopathology even within the relatively narrow age range of early childhood (18–71 months) (Wakschlag et al., 2010). These findings suggest that some child behavior problems might not be informative in caregiver‐report of psychopathology for children of different ages. Therefore, normative ranges of child behaviors should be clarified by research, and care should be taken when administering identical item sets across age groups. Moreover, our sensitivity analyses showed that the DIF associated with child race cannot be fully accounted for by SES variables (caregiver education and income), indicating a need for further investigation of potential measurement bias associated with race (e.g., race‐related cultural differences, trauma experiences).

In terms of measuring *Internalizing problems,* items with the most measurement bias were from the *Anxious/Depressed* and *Emotionally Reactive* subscales. Studies on anxiety and depression in young children have repeatedly emphasized the challenge of generalizing adult diagnostic criteria to young children (Carter et al., 2004; Luby et al., 2002; Tandon et al., 2009), and relying heavily on caregivers to infer the internal states (e.g., feelings) of young children. As for *Externalizing,* almost half of the items showed impactful DIF (10 out of 24), with three out of five items on the *Attention Problems* scale showing high DIF. Removing a large amount of high‐DIF items to construct the robust item set might result in changes in the construct validity of the *Externalizing* scale. *Externalizing* items with the most DIF are adjectives that may imply character judgment (e.g., *defiant, stubborn*). Taken together, many items with impactful DIF lack clear descriptions of observable behaviors, which might be more susceptible to bias driven by child and caregiver characteristics. Thus, measurement of early behavior problems may be enhanced by carefully operationalizing specific behaviors relevant to constructs under investigation to reduce the risk of bias (Merrell, 2001).

### Implications for using the CBCL/1.5–5

Given the large amount of DIF identified in our analyses of CBCL/1.5 items, it is important to consider the effects of measurement bias when using the CBCL/1.5–5. Practically, DIF could be handled in two ways: (a) by applying group‐specific parameters for score calculation, or (b) by removing items with impactful DIF. Given the technical requirements to apply group‐specific algorithms, we recommend CBCL/1.5–5 consumers follow the below guideline to administer the robust item sets with reduced DIF: (1) administer robust item sets listed in Table 3; (2) calculate summed scores for the desired domain; (3) use the crosswalk table on OSF repository for this study to convert robust item set sum scores to approximate full item set sum scores; (4) convert sum scores to T scores using CBCL score conversion tables. Researchers and clinicians can then apply the recommended cut‐offs based on the CBCL manual: 60–63 as borderline range, and above 63 as the clinical range. Changes in construct validity notwithstanding, the resulting T scores are expected to be comparable to the T score from the original CBCL but with fewer biases across groups that differ on the child and caregiver characteristics detailed above.

### Limitations and strengths

We only highlighted the main takeaways of our study and did not present all results in full detail in the manuscript. Decisions on how to winnow these results were made with attention to presentability while maintaining statistical rigor and transparency. First, although different decisions, such as the threshold of “impactful” DIF, could have been made, we believe the core conclusions would remain the same. We share item‐level parameter estimates and standard errors via OSF Repository for interested readers to conduct their own queries. Second, we had chosen to focus on the broad domains of *Internalizing*, *Externalizing*, and *Total Problems*. Thus, findings should not be generalized to the syndrome scales or researcher‐defined item subsets (MI/DIF analyses for syndrome scales are included in Appendix S2, given the emphasis here on broadband domains). Additionally, the current analysis could not distinguish between uniform DIF (i.e., DIF affecting all items in the same way) and differences in latent mean and variance. Future work including multiple raters can help clarify this distinction. Finally, the current analysis did not examine item discrimination and severity based on the criteria of clinical concerns due to the lack of such information, so robust item sets are not optimized for identifying clinical‐range problems.

Despite the abovementioned limitations, our study has many methodological strengths, including an unprecedently large and diverse sample from across the United States, with high representation across race, language, caregiver education, and income. Furthermore, the large sample size allowed us to perform analyses with many subdivisions of the data, yielding a highly multifaceted assessment of MI/DIF. The multivariate examinations also revealed the differential impact of certain variables in the presence of possible confounders. Relatedly, the application of the alignment approaches to MI/DIF allowed the efficient investigations of large numbers of groups and items while accommodating mismatches between item sets and observed item categories across groups.

### Conclusions

The CBCL/1.5–5 is a commonly used measure of early childhood behavior problems and risk for psychopathology. This study systematically applied a rigorous method to examine MI/DIF in a large, diverse sample and found measurement bias related to language version, caregiver education level and sex, and child age. Our identification of robust item sets with the least DIF that offer similar levels of information as the full item sets and could be applied across diverse samples to reduce measurement bias of the CBCL/1.5–5. Future work in child assessment should carefully consider the bias of caregiver‐report measures and devise methods to account for measurement bias when possible.

## AUTHOR CONTRIBUTIONS

**Shuting Zheng**: Conceptualization; Investigation; Methodology; Project administration; Resources; Supervision; Writing – original draft; Writing – review & editing. **Maxwell Mansolf**: Formal analysis; Methodology; Visualization; Writing – original draft; Writing – review & editing. **Monica McGrath**: Data curation; Supervision; Writing – review & editing. **Marie L. Churchill**: Data curation; Formal analysis; Project administration; Writing – review & editing. **Traci A. Bekelman**: Data curation; Funding acquisition; Writing – review & editing. **Patricia A. Brennan**: Data curation; Funding acquisition; Writing – review & editing. **Amy E. Margolis**: Data curation; Funding acquisition; Writing – review & editing. **Sara S. Nozadi**: Data curation; Writing – review & editing. **Theresa M. Bastain**: Data curation; Writing – review & editing. **Amy J. Elliott**: Data curation; Funding acquisition; Writing – review & editing. **Kaja Z. LeWinn**: Data curation; Funding acquisition; Writing – review & editing. **Julie A. Hofheimer**: Data curation; Funding acquisition; Writing – review & editing. **Leslie D. Leve**: Data curation; Funding acquisition; Writing – review & editing. **Brandon Rennie**: Data curation; Writing – review & editing. **Emily Zimmerman**: Data curation; Writing – review & editing. **Carmen A. Marable**: Data curation; Writing – review & editing. **Cindy T. McEvoy**: Data curation; Funding acquisition; Writing – review & editing. **Chang Liu**: Data curation; Writing – review & editing. **Alexis Sullivan**: Data curation; Writing – review & editing. **Tracey J. Woodruff**: Data curation; Funding acquisition; Writing – review & editing. **Samiran Ghosh**: Data curation; Funding acquisition; Writing – review & editing. **Bennett Leventhal**: Data curation; Funding acquisition; Writing – review & editing. **Assiamira Ferrara**: Data curation; Funding acquisition; Writing – review & editing. **Johnnye Lewis**: Data curation; Funding acquisition; Writing – review & editing. **Somer Bishop**: Conceptualization; Investigation; Supervision; Writing – review & editing.

## CONFLICT OF INTEREST STATEMENT

The authors have declared that they have no competing or potential conflicts of interest.

## ETHICAL CONSIDERATIONS

Study protocols of individual cohorts in the ECHO program were reviewed and approved by site‐specific institutional review boards. Participants provided informed consent for themselves and their children to be in the studies and have their data be shared for research use in the ECHO program.

## Supporting information

Supplementary Material

## Data Availability

The data that support the findings of this study are available from NIH ECHO program to ECHO‐affiliated researchers.
