# Supplementary material for: Measurement bias in caregiver‐report of early childhood behavior problems across demographic factors in an ECHO‐wide diverse sample
Source: JCPP Adv. 2023 Sep 20;4(1):e12198. doi: 10.1002/jcv2.12198 (PMC10933609; doi:10.1002/jcv2.12198)
Supplement: Supplementary file 1 — Supplementary Material [file JCV2-4-e12198-s001.docx]

Supporting Information

Table of Contents

[Appendix S1 Technical Description of Methods 3](#_Toc142987053)

[Statistical Analyses 3](#_Toc142987054)

[Configural Invariance Testing 3](#_Toc142987055)

[Alignment Method for MI/DIF Estimation 3](#_Toc142987056)

[ANOVA tests of MI/DIF 4](#_Toc142987057)

[Assessing the Impact of MI/DIF 8](#_Toc142987058)

[Appendix S2 Supplementary Analyses 10](#_Toc142987059)

[Additional Groupings and Domains 10](#_Toc142987060)

[Results and Discussion 12](#_Toc142987061)

[Model Fit 12](#_Toc142987062)

[Significance and Magnitude of DIF 12](#_Toc142987063)

[Decomposition of DIF in Multivariate Groupings 12](#_Toc142987064)

[Supplemental Tables and Figures 13](#_Toc142987065)

[Table S1 *Item frequencies for full analysis sample* 13](#_Toc142987066)

[Table S2 *Median Effect Sizes (eta-squared) of Main and Interaction Effects Related to Race on DIF* 14](#_Toc142987067)

[Table S3 *Median Effect Sizes (eta-squared) of Main and Interaction Effects Related to Language Version on DIF* 15](#_Toc142987068)

[Table S4 *Correlation and Mean Differences Between Linked Score Using the Robust Item Sets and Raw Summed Scores Using the Full Item Sets* 16](#_Toc142987069)

[Table S5 *Group Comparisons of T-scores of Full Item Set across Child and Caregiver Characteristics across Domains* 17](#_Toc142987070)

[Table S6 *Items without Any Significant DIF across Domains and Characteristics – Extra Language Groupings Only, Broadband Domains* 19](#_Toc142987071)

[Table S7 *Items without Any Significant DIF across Domains and Characteristics – Manuscript Groupings, Syndrome Scales* 20](#_Toc142987072)

[Table S8 *Items without Any Significant DIF across Domains and Characteristics – Extra Language Groupings Only, Syndrome Scales* 21](#_Toc142987073)

[Table S9 *Median Effect Sizes (eta-squared) of Main and Interaction Effects on DIF: Manuscript Groupings, Syndrome Scales* 22](#_Toc142987074)

[Table S10 *Median Effect Sizes (eta-squared) of Main and Interaction Effects on DIF: Race Groupings, Syndrome Scales* 23](#_Toc142987075)

[Table S11 *Median and Maximum Effect Sizes (eta-squared) of Main and Interaction Effects on DIF: Extra Language Groupings, Syndrome Scales* 24](#_Toc142987076)

[Figure S1 *Median Across Waves of Focal Group UIDS of Each Item with Significant DIF in Each Wave* 25](#_Toc142987077)

[Figure S2 *Model Fit for Analysis Models – Extra Language Groupings Only, Broadband Domains* 27](#_Toc142987078)

[Figure S3 *Model Fit for Analysis Models – Manuscript Groupings, Syndrome Scales* 28](#_Toc142987079)

[Figure S4 *Model Fit for Analysis Models – Extra Language Groupings Only, Syndrome Scales* 29](#_Toc142987080)

[Figure S5 *Items with Significant DIF and UIDS > 0.1 – Extra Language Groupings Only, Broadband Domains* 30](#_Toc142987081)

[Figure S6 *Items with Significant DIF and UIDS > 0.1 – Manuscript Groupings, Syndrome Scales* 32](#_Toc142987082)

[Figure S7 *Items with Significant DIF and UIDS > 0.1 – Extra Language Groupings Only, Syndrome Scales* 34](#_Toc142987083)

[Figure S8 *Distribution of UIDS by Grouping and Latent Constructs – Extra Language Groupings Only, Broadband Domains* 36](#_Toc142987084)

[Figure S9 *Distribution of UIDS by Grouping and Latent Constructs – Manuscript Groupings, Syndrome Scales* 38](#_Toc142987085)

[Figure S10 *Distribution of UIDS by Grouping and Latent Constructs – Extra Language Groupings Only, Syndrome Scales* 40](#_Toc142987086)

# Appendix S1 Technical Description of Methods

## Statistical Analyses

Per the response frequencies (Supplemental Table S1), all items were right-skewed, which often yielded low cell frequencies in the categories indicating more problems (1 and 2 on the CBCL scoring metric). To yield more stable estimates, response options for items were recoded as follows: (1) if fewer than 10 responses of 2 were observed, 2s were recoded to 1s; (2) if, after this recoding, fewer than 10 responses of 1 were observed, the corresponding item was not included in IRT estimation for that group, and that item/group combination was not considered in subsequent DIF analysis. Across all subsamples, there were 351 instances of items (2.7% of all item-subsample combinations) that were not included in IRT estimation or DIF analysis; among remaining items, 3313 instances (30.3%) required combining 2s and 1s.

### Configural Invariance Testing

All factors in the bifactor models for Total Problems were specified as orthogonal and estimated via the EM algorithm. For all models, convergence was verified and the second-order derivative test was used to ensure standard errors could be estimated for all models.

### Alignment Method for MI/DIF Estimation

The alignment method first estimates the configural model in each subgroup separately, constraining the mean of the latent variable in each group as zero and the variance as one. Due to this constraint, group differences in latent mean and variance are reflected instead in the item parameters. After estimation, we used an analytic model transformation to establish *approximate* measurement invariance, or the very-near measurement invariance of most item parameters while others are allowed to differ across groups. The alignment method is superior to traditional multi-group methods in terms of model convergence, complexity, and computational cost due to not requiring any multiple group model estimation or iterative refitting. For bifactor models, we used the marginal item parameters (Toland, Sulis, Giambona, Porcu, & Campbell, 2017) for the general factor in alignment and subsequent DIF analysis, transforming both estimates and standard errors to be comparable to the unidimensional item parameters in Internalizing and Externalizing.

### ANOVA tests of MI/DIF

The alignment method yields parameter estimates and standard errors for all model parameters in the aligned models. Considering a hypothetical alignment across two groups, one could use a *t* test to test for significant differences in the item parameters, comparing the differences in the estimates to the estimated standard error of that difference. With more than two groups, or with multivariate groupings as in the current work, a similar one- or two-way analysis of variance (ANOVA) is possible, yielding tests of main effects and interactions and estimation of effect size (η^2^). Before continuing, note what this ANOVA is testing: rather than differences between means as in conventional ANOVA, ANOVA conducted on estimates and standard errors tests whether the variability between estimates is greater than would be expected by chance given the estimated sampling variance, calculated as the squared standard error, of the estimates.

In conventional ANOVA, individual-level data are used to calculate sums of squares, degrees of freedom, and consequently mean squares, test statistics, and effect size estimates. However, in alignment output, individual-level data are not available from which to estimate sums of squares, as tests are sought of differences in item parameter estimates which relate via a complex, analytically intractable way to the raw item response data. Instead, only the estimates and standard errors are available, but note that these constitute sufficient statistics for the ANOVA. Specifically, standard errors of estimates are analogous to measures of within-group variability in ANOVA, which is used in the calculation of *mean square within groups* (*MS_W_*). Variability in the estimates themselves, which can be calculated crudely by simply applying the formula for sample variance to the estimates, represents between-group variability analogous to the variability in the sample means (which are, of course, estimates themselves) in conventional ANOVA, and can be used to calculate *mean square between groups* (*MS_B_*). Once *MS_W_* and *MS_B_* are calculated, a test statistic can be formed and tested from their ratio, constituting our test of DIF for each item parameter.

Like any statistical analysis, this approach comes with limitations. Specifically, conventional ANOVA assumes of normality within each group, so in order to conduct ANOVA analytically on estimates and standard errors we must assume normal sampling distributions for the item parameters. Posterior distributions of item parameters were not readily available from *mirt*, so this assumption was not tested, but the sample size requirement of *n* = 100 within each group, which was often satisfied many times over, suggests that this assumption was not severely violated. In addition, due to the aforementioned intractability of the relationship between the individual observations and the obtained estimates, the math below is consistent with one-way ANOVA of equally sized groups, implicitly assuming equal reliability of estimation for all groups.

One-Way ANOVA. Consider a one-way ANOVA test of one item parameter across levels of a single grouping variable, for example sex or age groupings. Let *e*_1_*, e*_2_, …, *e_G_* represent a set of estimates of the same item parameter, for example the item slope in the graded response model, across groups after alignment, where *G* represents the number of groups of the grouping variable of interest. Further, let *s*_1_*, s*_2_, …, *s_G_* represent the standard error estimates associated with *e*_1_*, e*_2_, …, *e_G_*.

Keeping to the analogy with one-way ANOVA, consider each estimate *e_g_* and standard error *s_g_*, *g* = 1, …, *G*, as defining a sampling distribution, conditional on the observed data, for the parameter of interest. Then, each squared standard error represents the *expected* squared deviation of any given sample estimate from the mean *e_g_* of the aforementioned sampling distribution. As in conventional ANOVA, these deviations can be used to calculate a summary statistic, which we refer to as *sum of squares within groups* (*SS_W_*), consistent with ANOVA nomenclature. Specifically, *SS_W_* can be calculated as

$${SS}_{W}=\sum_{g=1}^{G} s_{g}^{2}$$

The associated degrees of freedom *df_W_* is the number of groups *g*, rather than *g* – 1, because all that is needed to take an average squared standard error is to divide by the number of estimates used. In conventional ANOVA, the number of groups is included in the calculation of *df­_W_*, but only to obtain unbiased estimates of variability; here, variability estimates are available, so a simple average is sufficient. Dividing *SS­_W_* by *df_W_* gives *MS_W_*, an estimate of the variability in between-group estimates of the item parameter of interest that would be expected by chance alone based on the within-group standard errors obtained from IRT estimation and alignment.

As in conventional ANOVA, *sums of squares between groups* can be calculated as the sum of squared differences between the individual estimates and their mean:

$${SS}_{B}=\sum_{g=1}^{G} \left( e_{g}-\frac{\sum_{g=1}^{G} e_{g}}{G} \right)^{2}$$

Also as in conventional ANOVA, the associated degrees of freedom *df_B_* is calculate as *G* – 1, such that the formula for *MS_B_* = *SS_B_*/*df_B_* becomes that of the sample estimate of the variance when the item parameter estimates are treated as observations. The ratio *MS_B_*/*MS_W_* can then be referred to an *F* distribution with *df_B_* and *df_W_* degrees of freedom. However, this is not optimal because, while *MS­_W_* is calculated based on a limited number of standard errors of estimates, the denominator degrees of freedom is a complex function of sample size, number of items and groups, etc. To simplify this complexity, we instead compared *MS_B_/MS_W_* to an *F* distribution with *df_B_* and infinite degrees of freedom or, equivalently, compared *SS_B_*/*MS_W_* to a *χ*^2^ distribution with *df_B_* degrees of freedom.

Two-Way ANOVA. For multivariate groupings, which here were exclusively bivariate, a two-way ANOVA analogous to the one-way ANOVA above is needed. The formulas for *SS_W_*, *df_W_*, and *MS_W_* are identical to those above, except that each group *g* is defined by the intersection of levels of two grouping variables *A* and *B*, containing *G_A_* and *G_B_* levels each, such that, for a fully crossed design (i.e., one in which all groups have sufficient sample size to yield estimates and standard errors), *G* = *G_A_* * *G_B_*.

As in conventional two-way ANOVA, sums of squares for the main effect of *A* (*SS_GA_*) can be calculated as the sum of squared differences between the individual estimates and the mean estimate for the corresponding level of *A*:

$${SS}_{GA}=\sum_{g=1}^{G} \left( e_{g}-\frac{\sum_{g_{A}}^{G_{A}} e_{g_{A}}}{G_{A}} \right)^{2}$$

and likewise for similarly-defined *SS_GB_*. As in conventional two-way ANOVA, the associated degrees of freedom is the corresponding number of groups minus one (*df_GA_* = *G_A_* – 1; *df_GB_* = *G_B_* – 1). Sum of squares for the interaction *SS_GAB_* is also defined as in conventional two-way ANOVA:

$${SS}_{GAB}=\sum_{g=1}^{G} \left( e_{g}-\frac{\sum_{g_{A}}^{G_{A}} e_{g_{A}}}{G_{A}}-\frac{\sum_{g_{B}}^{G_{B}} e_{g_{B}}}{G_{B}}+\frac{\sum_{g=1}^{G} e_{g}}{G} \right)^{2}$$

with *SS_GAB_* = *SS_GA_* * *SS_GB_*. As in the one-way ANOVA case, for each mean square *MS_*_* for *A*, *B*, and their interaction, the ratio *MS_*_/MS_W_* to an *F* distribution with *df_*_* and infinite degrees of freedom or, equivalently, *SS_*_*/*MS_W_* can be compared to a *χ*^2^ distribution with *df_*_* degrees of freedom.

### Assessing the Impact of MI/DIF

As described in Meade (2010), UIDS is a comparison of scores estimated using parameters from the *focal group*, for which we want to estimate the impact of DIF; and the *baseline group*, against which we can compare the focal group. Differences in scores between these groups reflect the impact of differences in the relationship between the latent variable and item responses on item scores, and can be interpreted on the metric of the raw item responses (here, 0-2 for CBCL items). Here, because we had no *a priori* “baseline group” for all groupings, we calculated UIDS for each group multiple times within each grouping, each time with a different other group in the grouping serving as the baseline group. For example, for the age grouping, UIDS for the 18-to-27 month-old group was calculated by separately estimating UIDS while treating each other age group (27-36, 36-45, 45-54, and 54-72 months), resulting in four UIDS values for each item estimated in the 18-to-27 month-old group. These four UIDS values were then averaged to yield the UIDS value, representing the average difference between expected item responses across all groups other than the group for which UIDS is calculated. UADS was then calculated as the average of UIDS values across all items for the corresponding group, used to assess DIF. To obtain latent variable values we could use to compare expected scores, we combined latent trait estimates from the aligned models across all groups within a grouping, such that UIDS values reflected only DIF and not also differences in latent distribution between the groups.

Borrowing some notation from Meade (2010), let *ES­_si,F_*_|_*_θ_* represent the expected score for subject *s* for a given item *i* and for a given latent trait value *θ*, calculated using aligned item parameters from the focal group, and likewise let *EIS­_i,B_*_|_*_θ_* represent the expected score for subject *s* for a given item *i* and for a given latent trait value *θ*, calculated using aligned item parameters from the baseline group. Estimates of *θ* were obtained as the estimated factor scores in each group based on the aligned model for that group. Then, for each item in each group within each wave, UIDS*_i_* for each item *i* is calculated as the mean absolute difference between expected scores in the focal and baseline groups (Meade, 2010, equation 4):

$${UIDS}_{i}=\frac{\sum_{s=1}^{N} \left| {ES}_{si,F|\hat{\theta}}-{ES}_{si,B|\hat{\theta}} \right|}{N}$$

Items with higher UIDS*_i_* have a higher magnitude of DIF between the focal and baseline groups than items with lower UIDS*_i_*. Additionally, UIDS*_i_* is on the metric of the raw score, making it easy to interpret; for example, in the CBCL, a UIDS*_i_* value of 1 would indicate that DIF renders expected scores for the same latent trait value a full point different, on average, depending on group membership. In the CBCL, this would constitute a massive DIF effect because the items themselves range from 0 to 2, with most being heavily right-skewed (Achenbach & Rescorla, 2001, Figure 10).

We evaluated differential *test* functioning using the *signed test difference in the sample* (*STDS*) statistic, also described in Meade (2010). STDS is calculated as the sum, across items within each domain, of *SIDS_i_*, or the *signed* item difference in the sample for item *i*. *SIDS_i_* is calculated identically to *UIDS_i_* except without the absolute value:

$${SIDS}_{i}=\frac{\sum_{s=1}^{N} {ES}_{si,F|\hat{\theta}}-{ES}_{si,B|\hat{\theta}}}{N}$$

Note that *UIDS_i_* is not simply |*SIDS_i_*| because the absolute values are taken before the summation. Once *SIDS_i_* is calculated for each item *i*, *STDS* for the domain of interest is calculated as, for a domain with *I* items,

$${STDS}_{i}=\sum_{i=1}^{I} {SIDS}_{i}.$$

# Appendix S2 Supplementary Analyses

## Additional Groupings and Domains

To supplement the analyses reported in the main manuscript, we conducted supplemental analyses to repeat the presented measurement modeling and DIF analysis with multivariate groupings of language version with bilingual status and two-category caregiver education level. In addition, we conducted analyses with the main groupings and multivariate language version groupings separately for syndrome scales. In these analyses, our main interest was in the generalizability of results from the main analysis to these additional groupings and syndrome scales; however, given the supplemental nature of these analyses, further inspection of these results may reveal features not described here. In general, these analyses proceeded identically to the main analyses. Exceptions are described below.

Configural model estimation proceeded with language version multivariate groupings similarly to the main manuscript analyses but with different subsamples. Estimation proceeded identically for the syndrome scales as for internalizing or externalizing, with the corresponding items defining unidimensional models. The same multivariate tests of DIF and effect size determinations were conducted for multivariate language version and all grouping variables as for the multivariate groupings in the main manuscript (Tables 4 and S2).

“Robust” item sets for syndrome scales were constructed using the robust item sets for the corresponding broadband domains (Table 3); specifically, for syndrome scales which are components of Internalizing or Externalizing, robust item sets were constructed using the subset of items in the corresponding broadband domain in Table 3 (e.g., *Externalizing* for *Aggressive Behavior*) contained in that syndrome scale, while for *Sleep Problems* and *Other Problems*, robust item sets were constructed by identifying the subset of robust items in *Total Problems* contained therein. We opted for this definition, rather than re-deriving robust item sets for each syndrome scale, due to the increased psychometric stability of the broadband measures which enables more accurate assessment of DIF by virtue of the larger number of items. Crosswalk tables for robust item sets for syndrome scales are also included in the OSF repository alongside those for broadband domains; note that two syndrome scales, namely Withdrawn and Somatic Complaints, had all items contained in the robust item set for the respective broadband domain (Internalizing), such that no crosswalk is needed. Lastly, we note that researchers interested in constructing their own robust item sets based on our results can use the included results, and those in the OSF repository, to do so.

## Results and Discussion

### Model Fit

Model fit for supplemental configural models (Figures S2-S4) resembled that of analysis (A) (Figure 1), with most SRMSR below .08 and very few above .1, demonstrating acceptable model fit for these additional groupings and for syndrome scales.

### Significance and Magnitude of DIF

Lists of items without significant DIF, analogous to Table 2 from the manuscript, are displayed in Table S6-S8. Items with significant DIF and UIDS > .1 identified for multivariate language groupings (B) and broadband domains are displayed in Figure S5, and Figures S6-7 contains the same results for syndrome scales for original (A) and extra language groupings (B), which are analogous to Figure 2 from the manuscript. Jittered scatterplots of UIDS, noting items flagged as high-DIF items in the main analysis, are included in Figures S8-S10.

### Decomposition of DIF in Multivariate Groupings

Tables of eta squared decompositions of DIF for syndrome scales for original (A), race, and extra language (B) groupings are presented in Tables S9, S10, and S11, respectively.

# Supplemental Tables and Figures

## Table S1 *Item frequencies for full analysis sample*

| **Item Response** | | | | |
| --- | --- | --- | --- | --- |
| **Item** | **0** | **1** | **2** | **NA** |
| 1 | 8177 | 763 | 97 | 50 |
| 2 | 7871 | 1006 | 188 | 22 |
| 3 | 5944 | 2833 | 289 | 21 |
| 4 | 7286 | 1546 | 228 | 27 |
| 5 | 5739 | 2782 | 540 | 26 |
| 6 | 4620 | 3206 | 1043 | 218 |
| 7 | 6969 | 1685 | 403 | 30 |
| 8 | 3203 | 4268 | 1591 | 25 |
| 9 | 5485 | 2768 | 804 | 30 |
| 10 | 5271 | 3088 | 688 | 40 |
| 11 | 5699 | 2870 | 484 | 34 |
| 12 | 7726 | 1037 | 292 | 32 |
| 13 | 6635 | 2042 | 390 | 20 |
| 14 | 8717 | 292 | 50 | 28 |
| 15 | 5436 | 3171 | 418 | 62 |
| 16 | 4576 | 3641 | 834 | 36 |
| 17 | 7053 | 1713 | 296 | 25 |
| 18 | 7385 | 1418 | 247 | 37 |
| 19 | 8270 | 732 | 61 | 24 |
| 20 | 4823 | 3957 | 263 | 44 |
| 21 | 6837 | 1941 | 276 | 33 |
| 22 | 4643 | 2678 | 1739 | 27 |
| 23 | 5820 | 2928 | 302 | 37 |
| 24 | 6918 | 1721 | 403 | 45 |
| 25 | 8143 | 827 | 85 | 32 |
| 26 | 8572 | 408 | 64 | 43 |
| 27 | 6470 | 2177 | 416 | 24 |
| 28 | 8321 | 676 | 68 | 22 |
| 29 | 5061 | 3425 | 582 | 19 |
| 30 | 5554 | 2815 | 678 | 40 |
| 31 | 8287 | 602 | 154 | 44 |
| 32 | 5961 | 2586 | 480 | 60 |
| 33 | 5589 | 2906 | 554 | 38 |
| 34 | 6948 | 1701 | 403 | 35 |
| 35 | 8391 | 579 | 87 | 30 |
| 36 | 4645 | 2907 | 1493 | 42 |
| 37 | 6243 | 2185 | 618 | 41 |
| 38 | 6527 | 2059 | 468 | 33 |
| 39 | 8878 | 145 | 20 | 44 |
| 40 | 5852 | 2881 | 312 | 42 |
| 41 | 8723 | 255 | 62 | 47 |
| 42 | 8111 | 882 | 54 | 40 |
| 43 | 8585 | 420 | 40 | 42 |
| 44 | 6347 | 2403 | 293 | 44 |
| 45 | 8805 | 221 | 24 | 37 |
| 46 | 8717 | 255 | 62 | 53 |
| 47 | 8421 | 555 | 67 | 44 |
| 48 | 7516 | 1429 | 99 | 43 |
| 49 | 8213 | 667 | 166 | 41 |
| 50 | 7690 | 1272 | 84 | 41 |
| 51 | 8665 | 327 | 41 | 54 |
| 52 | 8403 | 543 | 97 | 44 |
| 53 | 8416 | 567 | 66 | 38 |
| 54 | 6185 | 2378 | 492 | 32 |
| 55 | 8401 | 538 | 99 | 49 |
| 56 | 7727 | 1111 | 195 | 54 |
| 57 | 8847 | 121 | 58 | 61 |
| 58 | 6427 | 2222 | 375 | 63 |
| 59 | 3678 | 4086 | 1276 | 47 |
| 60 | 8105 | 746 | 191 | 45 |
| 61 | 6765 | 2062 | 221 | 39 |
| 62 | 8301 | 694 | 42 | 50 |
| 63 | 8587 | 345 | 110 | 45 |
| 64 | 5644 | 2815 | 579 | 49 |
| 65 | 7323 | 1182 | 474 | 108 |
| 66 | 6562 | 2005 | 480 | 40 |
| 67 | 8707 | 293 | 43 | 44 |
| 68 | 7307 | 1540 | 197 | 43 |
| 69 | 5980 | 2805 | 261 | 41 |
| 70 | 8103 | 789 | 149 | 46 |
| 71 | 8363 | 564 | 116 | 44 |
| 72 | 6945 | 1644 | 449 | 49 |
| 73 | 7045 | 1801 | 196 | 45 |
| 74 | 7955 | 798 | 282 | 52 |
| 75 | 8801 | 197 | 39 | 50 |
| 76 | 7240 | 1032 | 758 | 57 |
| 77 | 8148 | 794 | 91 | 54 |
| 78 | 8659 | 348 | 35 | 45 |
| 79 | 7960 | 957 | 121 | 49 |
| 80 | 8718 | 230 | 76 | 63 |
| 81 | 5788 | 2788 | 459 | 52 |
| 82 | 7327 | 1549 | 159 | 52 |
| 83 | 8237 | 669 | 107 | 74 |
| 84 | 6929 | 1892 | 218 | 48 |
| 85 | 4775 | 3539 | 731 | 42 |
| 86 | 8004 | 889 | 147 | 47 |
| 87 | 8292 | 686 | 60 | 49 |
| 88 | 6405 | 2455 | 174 | 53 |
| 89 | 8819 | 192 | 22 | 54 |
| 90 | 8816 | 215 | 8 | 48 |
| 91 | 6911 | 1673 | 453 | 50 |
| 92 | 7872 | 993 | 164 | 58 |
| 93 | 8787 | 219 | 35 | 46 |
| 94 | 7195 | 1488 | 360 | 44 |
| 95 | 7806 | 1081 | 149 | 51 |
| 96 | 4547 | 3383 | 1102 | 55 |
| 97 | 4774 | 3657 | 600 | 56 |
| 98 | 8459 | 493 | 68 | 67 |
| 99 | 8243 | 708 | 68 | 68 |

## Table S2 *Median Effect Sizes (eta-squared) of Main and Interaction Effects Related to Race on DIF*

|  | **Term** | **Internalizing** | | **Externalizing** | | **Total Problems** | |
| --- | --- | --- | --- | --- | --- | --- | --- |
|  |  | **Median** | **Max** | **Median** | **Max** | **Median** | **Max** |
| **Race-Education** | Race | 0.076 | 0.629 | 0.094 | 0.729 | 0.097 | 0.763 |
|  | Education | 0.219 | 0.793 | 0.243 | 0.725 | 0.276 | 0.858 |
|  | Race: Education | 0.112 | 0.586 | 0.118 | 0.465 | 0.112 | 0.545 |
| **Race-Income** | Race | 0.119 | 0.704 | 0.138 | 0.664 | 0.119 | 0.638 |
|  | Income | 0.155 | 0.576 | 0.114 | 0.515 | 0.159 | 0.723 |
|  | Race: Income | 0.173 | 0.439 | 0.204 | 0.444 | 0.191 | 0.570 |

## Table S3 *Median Effect Sizes (eta-squared) of Main and Interaction Effects Related to Language Version on DIF*

|  | **Term** | **Internalizing** | | **Externalizing** | | **Total Problems** | |
| --- | --- | --- | --- | --- | --- | --- | --- |
|  |  | **Median** | **Max** | **Median** | **Max** | **Median** | **Max** |
| **Language- Bilingual** | Bilingual | 0.092 | 0.530 | 0.048 | 0.386 | 0.061 | 0.743 |
|  | Language | 0.183 | 0.977 | 0.406 | 0.964 | 0.307 | 0.985 |
|  | Bilingual: Language | 0.023 | 0.548 | 0.072 | 0.552 | 0.069 | 0.676 |
| **Language-Education** | Education | 0.195 | 0.930 | 0.394 | 0.938 | 0.253 | 0.978 |
|  | Language | 0.144 | 0.641 | 0.069 | 0.841 | 0.105 | 0.844 |
|  | Education: Language | 0.094 | 0.672 | 0.079 | 0.682 | 0.078 | 0.608 |

## Table S4 *Correlation and Mean Differences Between Linked Score Using the Robust Item Sets and Raw Summed Scores Using the Full Item Sets*

| **Domain** | **Linked-Observed Correlation ^a^** | **Bias in Linked Scores ^b^** |
| --- | --- | --- |
| **Total Problems** | 0.989 | -0.004 |
| **Internalizing** | 0.963 | 0.008 |
| **Externalizing** | 0.965 | 0.007 |
| **Emotionally Reactive** | 0.952 | 0.028 |
| **Anxious/Depressed** | 0.653 | 0.142 |
| **Attention Problems** | 0.819 | 0.051 |
| **Aggressive Behavior** | 0.965 | 0.009 |
| **Sleep Problems** | 0.910 | 0.037 |

Note:

a. Bivariate correlations were calculated between (1) raw summed scores of all items for each domain in the original CBCL/1.5-5 and (2) the linked scores, from adding up the robust item set then using the crosswalk tables to link to the metric of the full domain.

b. Bias is calculated as the mean difference between the two sets of scores, which measures whether the linked scores are systematically higher or lower than the true scores on the full CBCL.

## Table S5 *Group Comparisons of T-scores of Full Item Set across Child and Caregiver Characteristics across Domains*

| **Grouping Variable** | **Group** | **N** | **Externalizing** | **Internalizing** | **Total Problems** |
| --- | --- | --- | --- | --- | --- |
| **Child Characteristics** | | | | | |
| **Age (months)** |  |  | ***F(4,7380)=22.340; p=0.000, η^2^=.012*** | ***F(4,7380)=19.570; p=0.000, η^2^=.010*** | ***F(4,7380)=20.762; p=0.000, η^2^=.011*** |
|  | [18,27) | 2241 | 44.92 (9.59) | 42.62 (9.52) | 43.90 (9.59) |
|  | [27,36) | 1328 | 46.60 (10.15) | 45.16 (10.32) | 46.23 (10.29) |
|  | [36,45) | 1155 | 46.06 (10.61) | 45.21 (10.65) | 45.70 (10.78) |
|  | [45,54) | 983 | 44.16 (10.30) | 44.10 (10.11) | 43.82 (10.16) |
|  | [54,72) | 1678 | 43.51 (10.26) | 44.47 (10.42) | 43.50 (10.31) |
| **Child Sex** |  |  | ***F(1,7381)=46.582; p=0.000, η^2^=.006*** | ***F(1,7381)=4.757; p=0.029, η^2^=.001*** | ***F(1,7381)=22.808; p=0.000, η^2^=.003*** |
|  | Female | 3527 | 44.14 (10.12) | 43.83 (10.19) | 43.91 (10.17) |
|  | Male | 3856 | 45.75 (10.15) | 44.35 (10.17) | 45.04 (10.20) |
| **Bilingual** |  |  | ***F(1,6507)=5.378; p=0.020, η^2^=.001*** | ***F(1,6507)=0.042; p=0.838, η^2^<.001*** | ***F(1,6507)=0.103; p=0.748, η^2^<.001*** |
|  | Bilingual | 2345 | 44.53 (10.33) | 44.01 (10.30) | 44.37 (10.38) |
|  | Not Bilingual | 4164 | 45.14 (10.12) | 43.96 (10.14) | 44.46 (10.16) |
| **NDD** |  |  | ***F(1,4491)=91.203; p=0.000, η^2^=.020*** | ***F(1,4491)=109.002; p=0.000, η^2^=.024*** | ***F(1,4491)=141.802; p=0.000, η^2^=.031*** |
|  | NDD | 552 | 49.35 (12.38) | 48.91 (12.02) | 49.83 (12.35) |
|  | No NDD | 3941 | 44.89 (9.95) | 44.12 (9.78) | 44.35 (9.78) |
| **Caregiver Characteristics** | | | | | |
| **Caregiver Depression** |  |  | ***F(1,4038)=136.734; p=0.000, η^2^=.033*** | ***F(1,4038)=98.500; p=0.000, η^2^=.024*** | ***F(1,4038)=149.327; p=0.000, η^2^=.036*** |
|  | HighDep | 247 | 52.67 (11.32) | 50.76 (11.18) | 52.66 (11.47) |
|  | LowDep | 3793 | 44.82 (10.15) | 44.06 (10.23) | 44.38 (10.23) |
| **Income** |  |  | ***F(4,6148)=35.107; p=0.000, η^2^=.022*** | ***F(4,6148)=46.758; p=0.000, η^2^=.030*** | ***F(4,6148)=53.524; p=0.000, η^2^=.034*** |
|  | <$30,000 | 1583 | 43.03 (9.33) | 41.98 (9.10) | 42.12 (8.94) |
|  | $30,000-$49,999 | 681 | 46.00 (10.70) | 44.79 (10.82) | 45.60 (10.88) |
|  | $50,000-$74,999 | 838 | 45.59 (9.85) | 43.99 (9.69) | 44.85 (9.83) |
|  | $75,000-$99,999 | 602 | 44.51 (9.74) | 42.97 (9.75) | 43.77 (9.52) |
|  | $100,000 or more | 2301 | 46.59 (10.93) | 46.24 (11.00) | 46.60 (11.25) |
| **Language Version** |  |  | ***F(1,6956)=4.738; p=0.030, η^2^=.001*** | ***F(1,6956)=6.055; p=0.014, η^2^=.001*** | ***F(1,6956)=12.717; p=0.000, η^2^=.002*** |
|  | English | 6193 | 44.75 (10.17) | 43.89 (10.10) | 44.20 (10.15) |
|  | Spanish | 765 | 45.59 (10.04) | 44.85 (10.82) | 45.59 (10.47) |
| **Maternal Education** |  |  | ***F(4,7227)=53.424; p=0.000, η^2^=.029*** | ***F(4,7227)=63.704; p=0.000, η^2^=.034*** | ***F(4,7227)=75.692; p=0.000, η^2^=.040*** |
|  | <high school | 545 | 43.89 (9.59) | 42.86 (9.73) | 43.24 (9.50) |
|  | High school | 1451 | 46.30 (10.45) | 45.65 (10.43) | 46.02 (10.66) |
|  | Some college | 1741 | 47.73 (11.10) | 47.81 (11.25) | 48.27 (11.56) |
|  | Bachelor’s degree | 1762 | 42.67 (8.99) | 41.68 (9.19) | 41.78 (8.73) |
|  | >=Master’s Degree | 1676 | 46.44 (10.67) | 45.16 (10.33) | 45.99 (10.56) |
| **Respondent Sex** |  |  | ***F(1,7383)=6.876; p=0.009, η^2^=.001*** | ***F(1,7383)=0.816; p=0.366, η^2^<.001*** | ***F(1,7383)=1.986; p=0.159, η^2^<.001*** |
|  | Female | 7061 | 44.91 (10.22) | 44.12 (10.25) | 44.46 (10.27) |
|  | Male | 324 | 46.43 (8.79) | 43.60 (8.70) | 45.28 (8.66) |

## Table S6 *Items without Any Significant DIF across Domains and Characteristics – Extra Language Groupings Only, Broadband Domains*

| **Latent Construct** | **Item** | **Content** | **Subdomain** |
| --- | --- | --- | --- |
| **Internalizing** | 07 | Can't Stand Things Out of Place | Somatic Complaints |
| **Total Problems** | 17 | Destroys Own Things | Other Problems |
|  | 57 | Eye Problems | Other Problems |
|  | 74 | Sleeps Little | Sleep Problems |
|  | 80 | Strange Behavior | Other Problems |

## Table S7 *Items without Any Significant DIF across Domains and Characteristics – Manuscript Groupings, Syndrome Scales*

| **Domain** | **Characteristic** | **Item** | **Content** |
| --- | --- | --- | --- |
| **Aggressive Behavior** | Child | 08 | Can't Stand Waiting |
|  |  | 27 | Lacks Guilt |
|  |  | 66 | Screams |
|  |  | 85 | Temper |
|  |  | 53 | Attacks People |
| **Anxious/Depressed** | Child | 43 | Looks Unhappy |
|  | Caregiver | 47 | Nervous |
|  |  | 51 | Panics |
| **Sleep Problems** | Child | 38 | Trouble Sleeping |
| **Withdrawn** | Child | 70 | Little Affection |
|  |  | 71 | Little Interest |
|  |  | 62 | Refuses Active Games |
|  | Caregiver | 70 | Little Affection |

## Table S8 *Items without Any Significant DIF across Domains and Characteristics – Extra Language Groupings Only, Syndrome Scales*

| **Domain** | **Item** | **Content** |
| --- | --- | --- |
| **Emotionally Reactive** | 21 | Disturbed by Change |
| **Sleep Problems** | 38 | Trouble Sleeping |
|  | 64 | Resists Bed |
| **Withdrawn** | 02 | Acts Too Young |

## Table S9 *Median Effect Sizes (eta-squared) of Main and Interaction Effects on DIF: Manuscript Groupings, Syndrome Scales*

|  | **Term** | **Aggressive Behavior** | | **Attention Problems** | | **Anxious/Depressed** | |
| --- | --- | --- | --- | --- | --- | --- | --- |
|  |  | **Median** | **Max** | **Median** | **Max** | **Median** | **Max** |
| **Education-Income** | Education | 0.058 | 0.548 | 0.090 | 0.215 | 0.056 | 0.420 |
|  | Income | 0.242 | 0.727 | 0.246 | 0.753 | 0.298 | 0.685 |
|  | Education: Income | 0.112 | 0.546 | 0.071 | 0.401 | 0.079 | 0.475 |
| **Sex-Age** | Age | 0.315 | 0.860 | 0.306 | 0.719 | 0.406 | 0.914 |
|  | Sex | 0.028 | 0.468 | 0.024 | 0.205 | 0.025 | 0.361 |
|  | Sex:Age | 0.129 | 0.438 | 0.116 | 0.325 | 0.140 | 0.409 |
| **Sex-NDD** | Sex | 0.091 | 0.508 | 0.076 | 0.492 | 0.067 | 0.218 |
|  | NDD | 0.095 | 0.783 | 0.307 | 0.622 | 0.322 | 0.528 |
|  | Sex:NDD | 0.070 | 0.504 | 0.035 | 0.162 | 0.126 | 0.319 |

|  | **Term** | **Emotionally Reactive** | | **Sleep Problems** | | **Somatic Complaints** | |
| --- | --- | --- | --- | --- | --- | --- | --- |
|  |  | **Median** | **Max** | **Median** | **Max** | **Median** | **Max** |
| **Education-Income** | Education | 0.034 | 0.232 | 0.035 | 0.619 | 0.059 | 0.313 |
|  | Income | 0.251 | 0.829 | 0.190 | 0.479 | 0.134 | 0.559 |
|  | Education: Income | 0.115 | 0.516 | 0.211 | 0.572 | 0.299 | 0.712 |
| **Sex-Age** | Age | 0.199 | 0.858 | 0.364 | 0.908 | 0.349 | 0.755 |
|  | Sex | 0.035 | 0.460 | 0.023 | 0.125 | 0.020 | 0.329 |
|  | Sex:Age | 0.212 | 0.504 | 0.121 | 0.270 | 0.219 | 0.547 |
| **Sex-NDD** | Sex | 0.066 | 0.434 | 0.040 | 0.373 | 0.077 | 0.475 |
|  | NDD | 0.269 | 0.761 | 0.094 | 0.644 | 0.072 | 0.785 |
|  | Sex:NDD | 0.066 | 0.284 | 0.214 | 0.463 | 0.164 | 0.589 |

|  | **Term** | **Withdrawn** | |
| --- | --- | --- | --- |
|  |  | **Median** | **Max** |
| **Education-Income** | Education | 0.0135 | 0.5563 |
|  | Income | 0.1579 | 0.4852 |
|  | Education:Income | 0.1249 | 0.4488 |
| **Sex-Age** | Age | 0.2903 | 0.7457 |
|  | Sex | 0.0337 | 0.2123 |
|  | Sex:Age | 0.208 | 0.4256 |
| **Sex-NDD** | Sex | 0.111 | 0.3022 |
|  | NDD | 0.0631 | 0.9054 |
|  | Sex:NDD | 0.0533 | 0.4146 |

## Table S10 *Median Effect Sizes (eta-squared) of Main and Interaction Effects on DIF: Race Groupings, Syndrome Scales*

|  | **Term** | **Aggressive Behavior** | | **Attention Problems** | | **Anxious/Depressed** | |  |
| --- | --- | --- | --- | --- | --- | --- | --- | --- |
|  |  | **Median** | **Max** | **Median** | **Max** | **Median** | **Max** | |
| **Race-Education** | Education | 0.094 | 0.680 | 0.022 | 0.356 | 0.143 | 0.430 | |
|  | Race | 0.225 | 0.735 | 0.166 | 0.513 | 0.174 | 0.677 | |
|  | Race: Education | 0.125 | 0.470 | 0.194 | 0.415 | 0.113 | 0.297 | |
| **Race-Income** | Income | 0.107 | 0.531 | 0.084 | 0.618 | 0.183 | 0.332 | |
|  | Race | 0.128 | 0.559 | 0.122 | 0.427 | 0.159 | 0.434 | |
|  | Race: Income | 0.185 | 0.424 | 0.198 | 0.414 | 0.185 | 0.281 | |

|  | **Term** | **Emotionally Reactive** | | **Sleep Problems** | | **Somatic Complaints** | |
| --- | --- | --- | --- | --- | --- | --- | --- |
|  |  | **Median** | **Max** | **Median** | **Max** | **Median** | **Max** |
| **Race-Education** | Education | 0.033 | 0.292 | 0.035 | 0.393 | 0.053 | 0.404 |
|  | Race | 0.284 | 0.638 | 0.272 | 0.592 | 0.120 | 0.461 |
|  | Race: Education | 0.085 | 0.473 | 0.150 | 0.586 | 0.229 | 0.539 |
| **Race-Income** | Income | 0.092 | 0.437 | 0.060 | 0.215 | 0.172 | 0.530 |
|  | Race | 0.127 | 0.421 | 0.138 | 0.603 | 0.054 | 0.377 |
|  | Race: Income | 0.189 | 0.579 | 0.252 | 0.437 | 0.124 | 0.396 |

|  | **Term** | **Withdrawn** | | | |  |
| --- | --- | --- | --- | --- | --- | --- |
|  |  | **Median** | | **Max** | |  |
| **Race-Education** | Education | | 0.069 | | 0.330 | |
|  | Race | | 0.211 | | 0.441 | |
|  | Race: Education | | 0.196 | | 0.265 | |
| **Race-Income** | Income | | 0.085 | | 0.375 | |
|  | Race | | 0.145 | | 0.335 | |
|  | Race: Income | | 0.197 | | 0.302 | |

## Table S11 *Median and Maximum Effect Sizes (eta-squared) of Main and Interaction Effects on DIF: Extra Language Groupings, Syndrome Scales*

|  | **Term** | **Aggressive Behavior** | | **Attention Problems** | | **Anxious/Depressed** | |
| --- | --- | --- | --- | --- | --- | --- | --- |
|  |  | **Median** | **Max** | **Median** | **Max** | **Median** | **Max** |
| **Bilingual-Language** | Bilingual | 0.033 | 0.373 | 0.121 | 0.248 | 0.048 | 0.510 |
|  | Language | 0.415 | 0.957 | 0.232 | 0.889 | 0.082 | 0.978 |
|  | Bilingual: Language | 0.057 | 0.531 | 0.100 | 0.708 | 0.077 | 0.578 |
| **Education-Language** | Language | 0.442 | 0.944 | 0.218 | 0.885 | 0.138 | 0.888 |
|  | Education | 0.074 | 0.797 | 0.098 | 0.377 | 0.152 | 0.869 |
|  | Education: Language | 0.065 | 0.512 | 0.077 | 0.291 | 0.103 | 0.473 |

|  | **Term** | **Emotionally Reactive** | | **Sleep Problems** | | **Somatic Complaints** | |
| --- | --- | --- | --- | --- | --- | --- | --- |
|  |  | **Median** | **Max** | **Median** | **Max** | **Median** | **Max** |
| **Bilingual-Language** | Bilingual | 0.075 | 0.493 | 0.040 | 0.412 | 0.194 | 0.538 |
|  | Language | 0.139 | 0.945 | 0.172 | 0.751 | 0.186 | 0.837 |
|  | Bilingual: Language | 0.059 | 0.316 | 0.026 | 0.520 | 0.045 | 0.608 |
| **Education-Language** | Language | 0.214 | 0.954 | 0.093 | 0.779 | 0.107 | 0.768 |
|  | Education | 0.044 | 0.422 | 0.041 | 0.215 | 0.150 | 0.686 |
|  | Education: Language | 0.098 | 0.198 | 0.099 | 0.544 | 0.045 | 0.498 |

|  | **Term** | **Withdrawn** | |
| --- | --- | --- | --- |
|  |  | **Median** | **Max** |
| **Bilingual-Language** | Bilingual | 0.060 | 0.412 |
|  | Language | 0.100 | 0.561 |
|  | Bilingual: Language | 0.072 | 0.359 |
| **Education-Language** | Language | 0.077 | 0.629 |
|  | Education | 0.056 | 0.392 |
|  | Education: Language | 0.048 | 0.181 |

## Figure S1 *Median Across Waves of Focal Group UIDS of Each Item with Significant DIF in Each Wave*


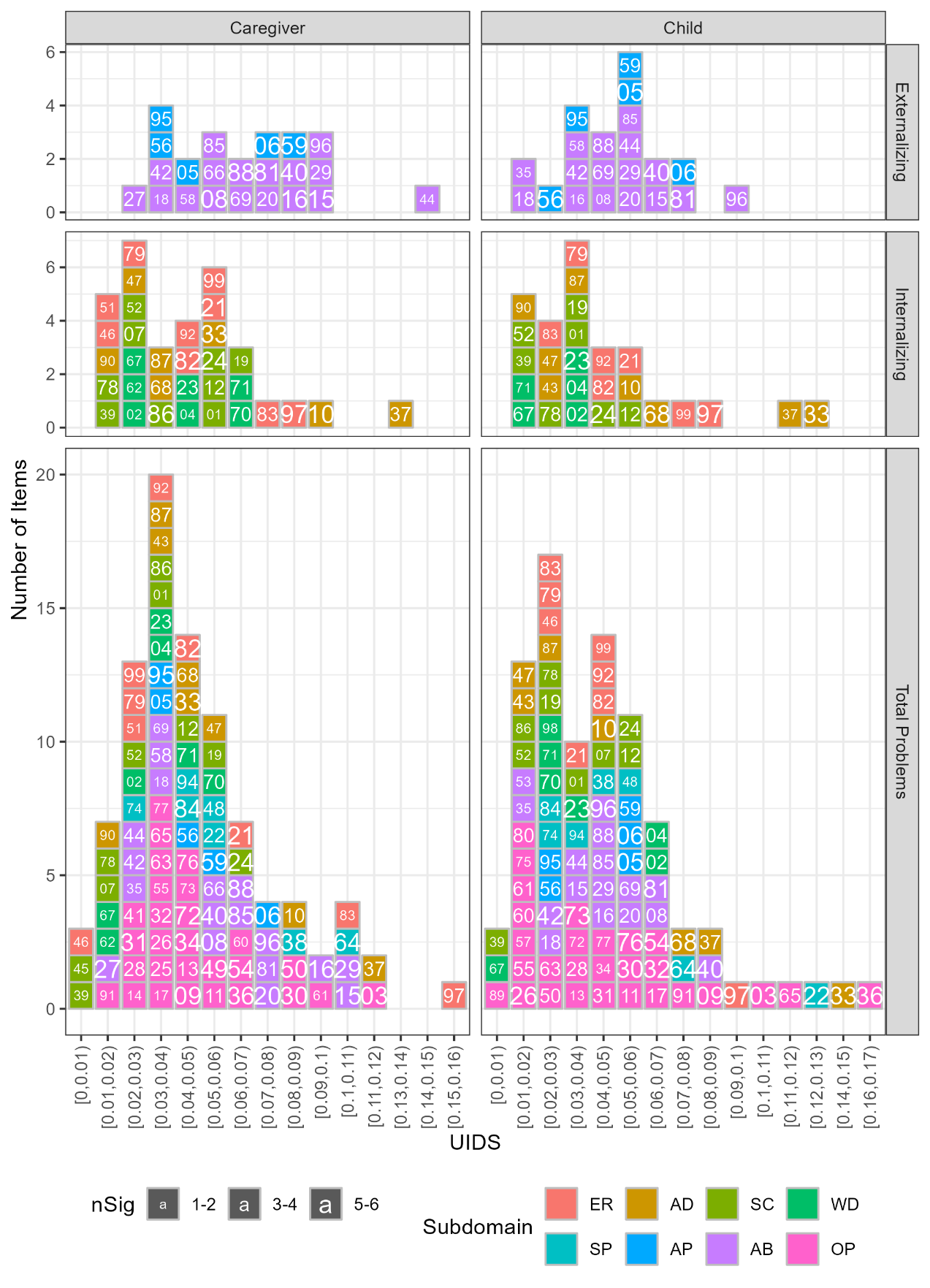


*Note*. *ER* = Emotionally Reactive; *AD* = Anxious/Depressed; *SC* = Somatic Complaints; *WD* = Withdrawn; *SP* = Sleep Problems; *AP* = Attention Problems; *AB* = Aggressive Behavior; *OP* = Other Problems

## Figure S2 *Model Fit for Analysis Models – Extra Language Groupings Only, Broadband Domains*


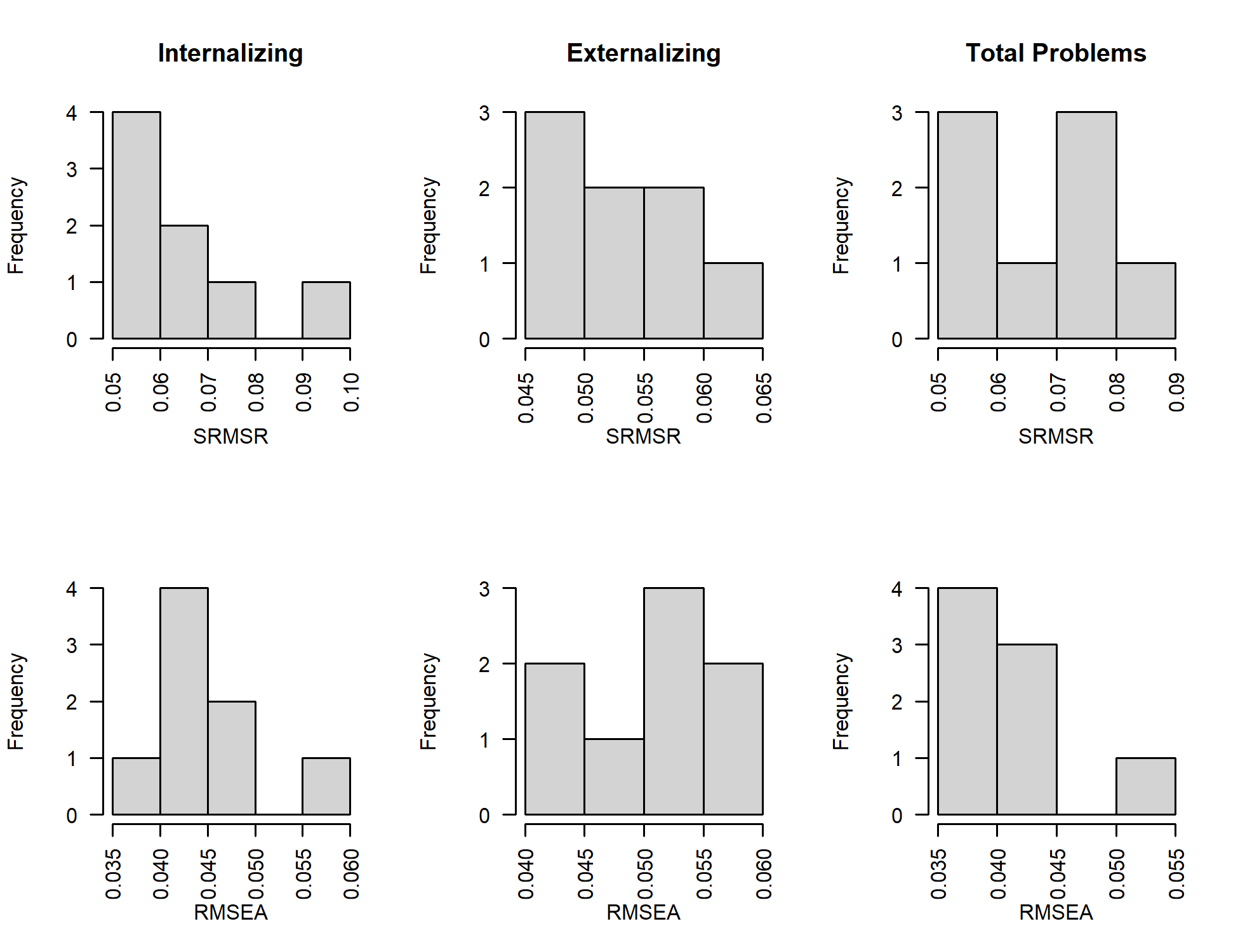


*Note*. *SRMSR* = Standardized Root Mean Squared Residual; *RMSEA* = Root Mean Squared Error of Approximation

## Figure S3 *Model Fit for Analysis Models – Manuscript Groupings, Syndrome Scales*


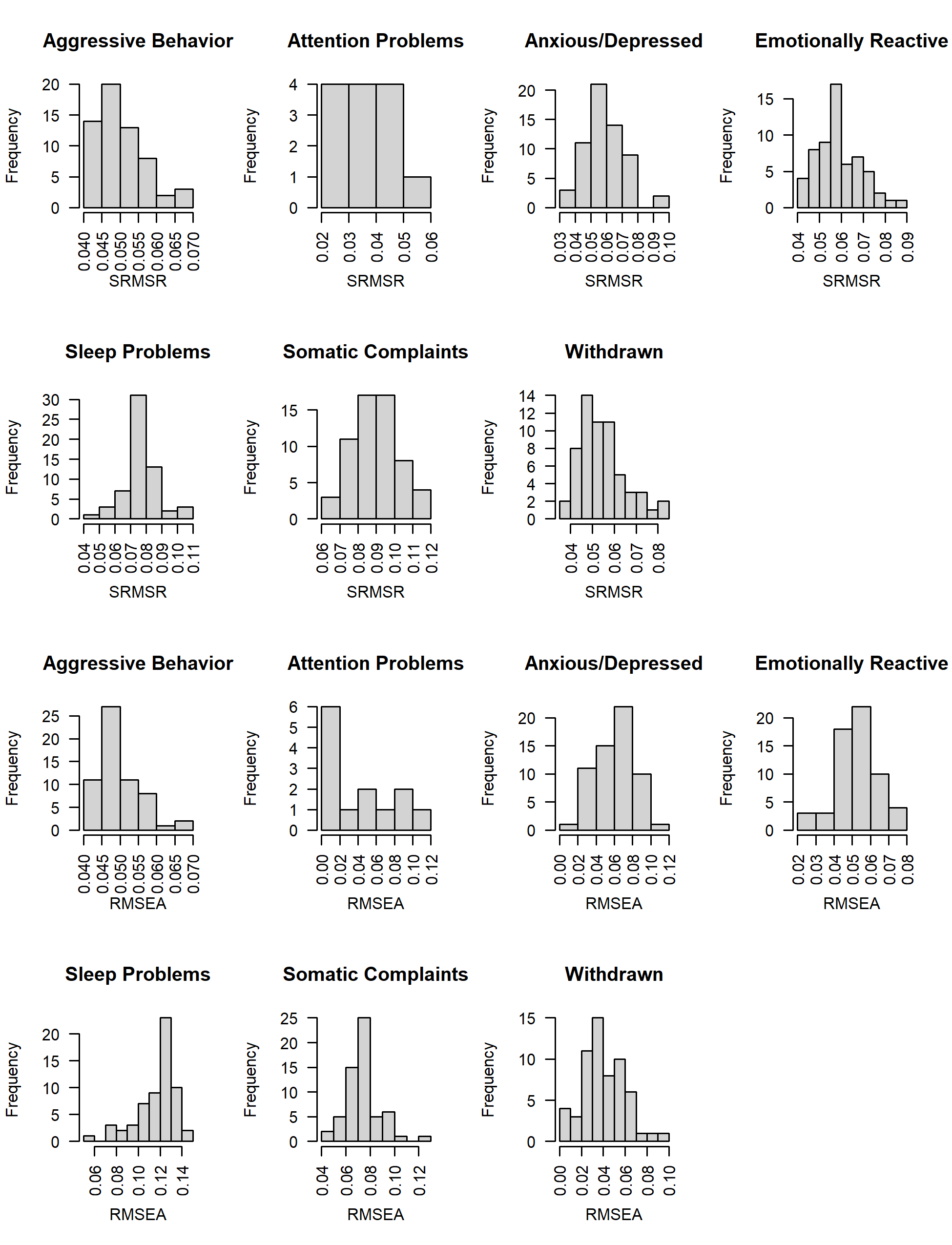


*Note*. *SRMSR* = Standardized Root Mean Squared Residual; *RMSEA* = Root Mean Squared Error of Approximation

## Figure S4 *Model Fit for Analysis Models – Extra Language Groupings Only, Syndrome Scales*


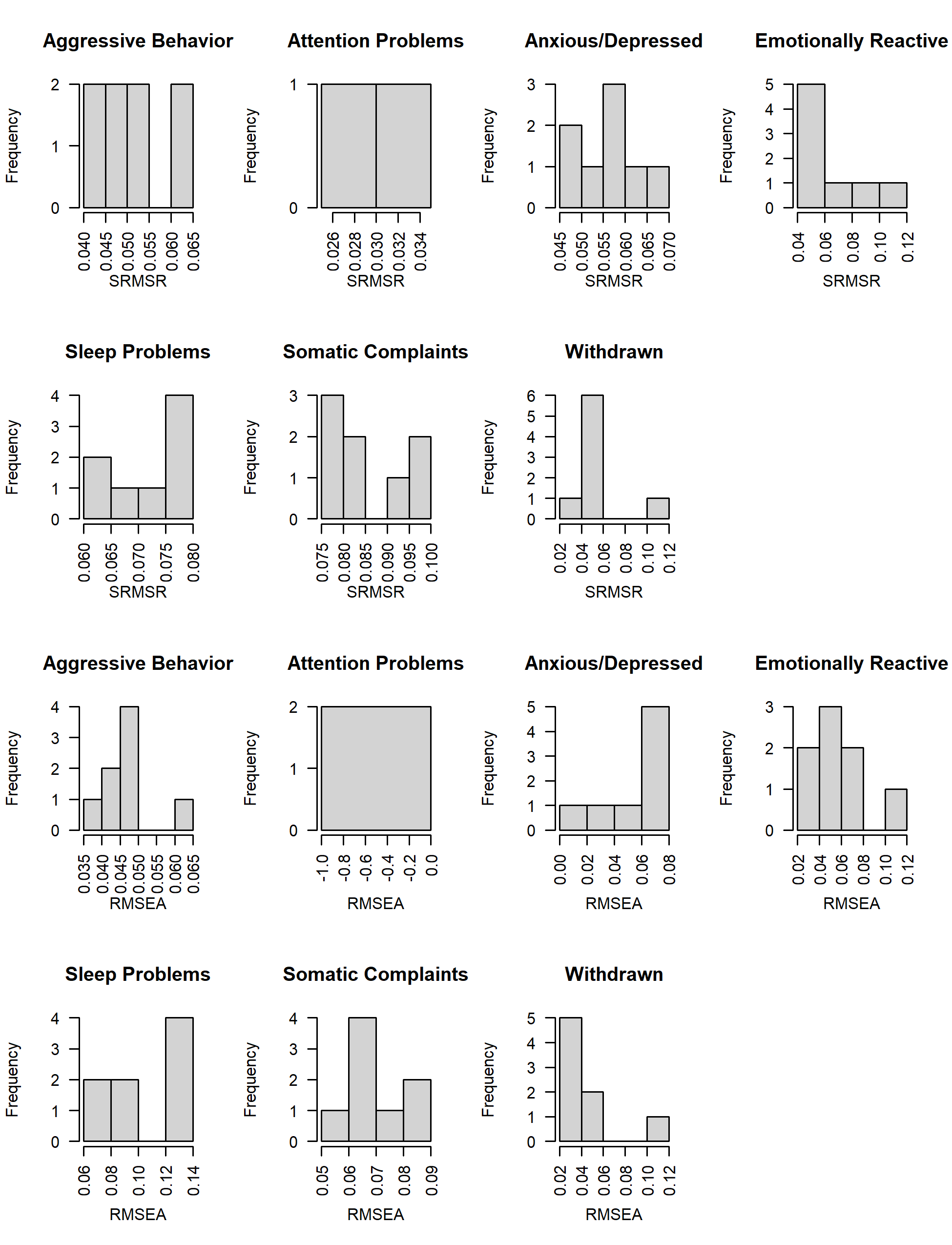


*Note*. *SRMSR* = Standardized Root Mean Squared Residual; *RMSEA* = Root Mean Squared Error of Approximation

## Figure S5 *Items with Significant DIF and UIDS > 0.1 – Extra Language Groupings Only, Broadband Domains*


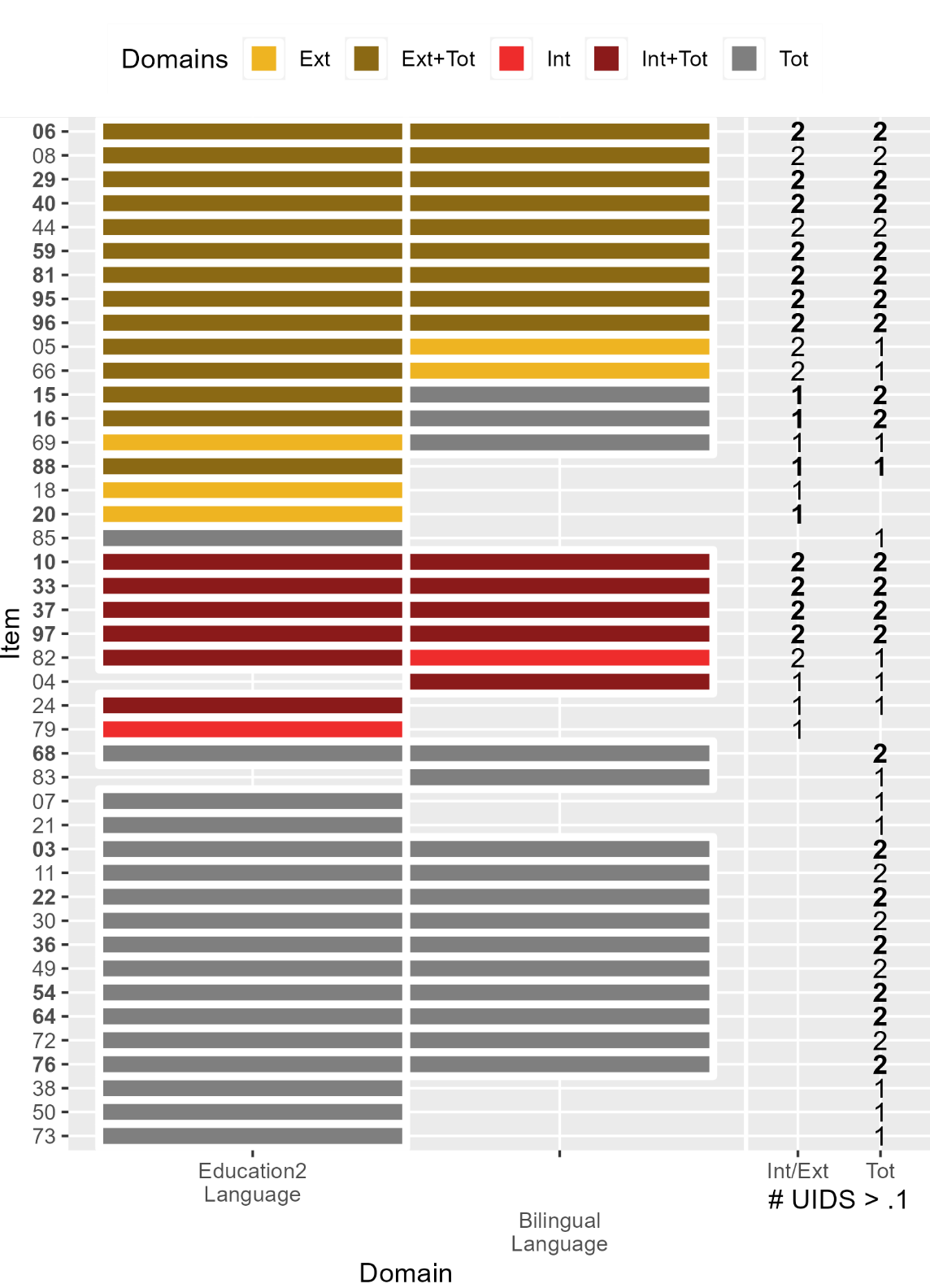


*Note*. *UIDS* = Unsigned Item Difference in the Sample; *Int* = Internalizing; *Ext* = Externalizing; *Tot* = Total Problems. Items with no significant DIF or no UIDS > .1 are excluded. Items are grouped by domains and sorted in decreasing order of the number of groupings with UIDS > .1 of items; groupings are sorted by the number of items with UIDS >.1 within the grouping. The numbers on the two right-hand columns show the number of groupings with UIDS>.1 for the specific items within domains.

## Figure S6 *Items with Significant DIF and UIDS > 0.1 – Manuscript Groupings, Syndrome Scales*


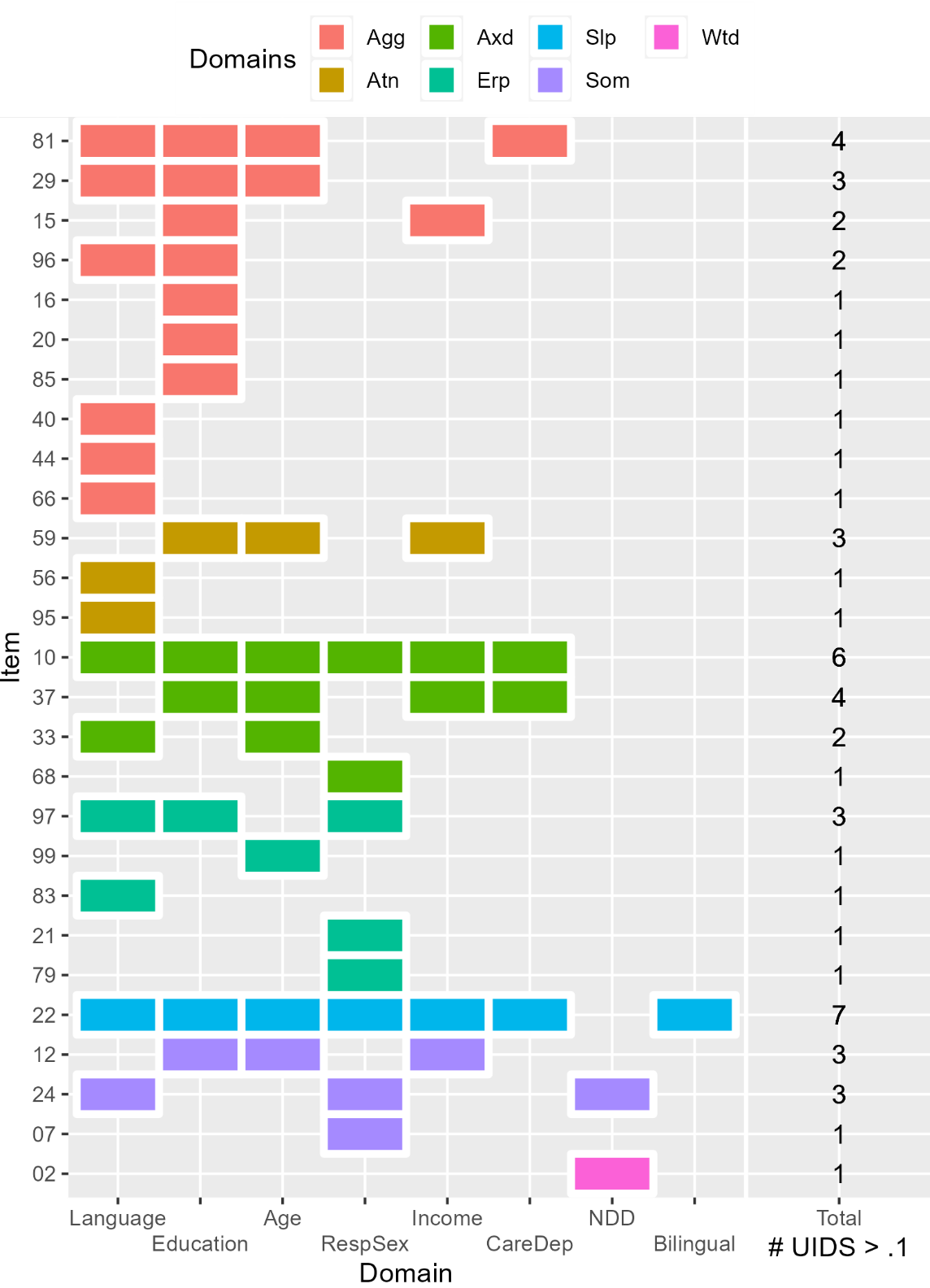


*Note*. *UIDS* = Unsigned Item Difference in the Sample; *RespSex* = Respondent Sex; *NDD* = Neurodevelopmental Disorder; *Agg* = Aggressive Behavior; *Atn* = Attention Problems; *Axd* = Anxious/Depressed; *Erp* = Emotionally Reactive; *Oth* = Other Problems; *Slp* = Sleep Problems; *Som* = Somatic Complaints; *Wtd* = Withdrawn. Items with no significant DIF or no UIDS > .1 are excluded. Items are grouped by domains and sorted in decreasing order of the number of groupings with UIDS > .1 of items. The numbers in the right-hand column show the number of groupings with UIDS>.1, i.e., the number of colored boxes in the corresponding row.

## Figure S7 *Items with Significant DIF and UIDS > 0.1 – Extra Language Groupings Only, Syndrome Scales*


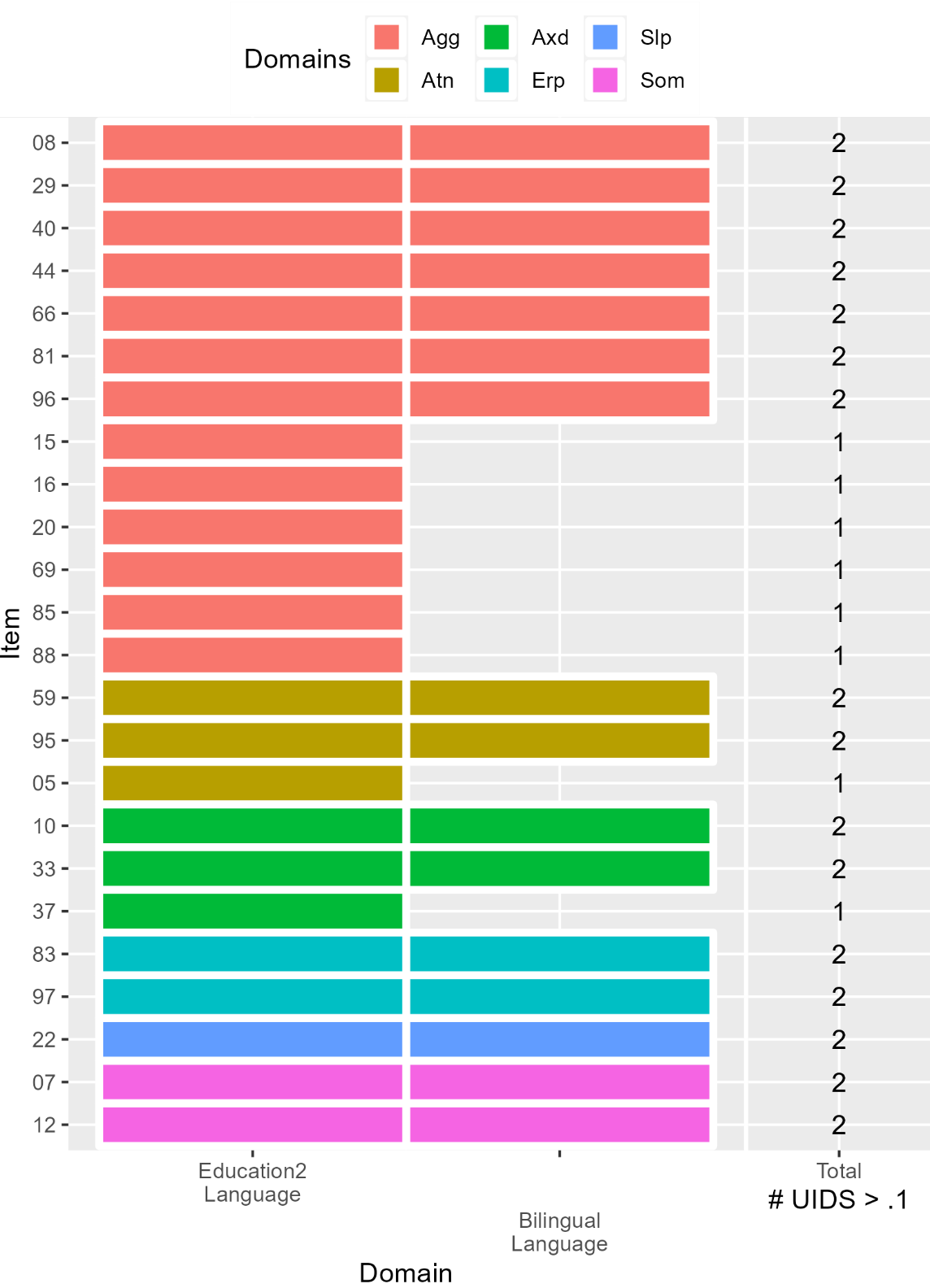


*Note*. *UIDS* = Unsigned Item Difference in the Sample; *RespSex* = Respondent Sex; *NDD* = Neurodevelopmental Disorder; *Agg* = Aggressive Behavior; *Atn* = Attention Problems; *Axd* = Anxious/Depressed; *Erp* = Emotionally Reactive; *Oth* = Other Problems; *Slp* = Sleep Problems; *Som* = Somatic Complaints. Items with no significant DIF or no UIDS > .1 are excluded. Items are grouped by domains and sorted in decreasing order of the number of groupings with UIDS > .1 of items. The numbers in the right-hand column show the number of groupings with UIDS>.1, i.e., the number of colored boxes in the corresponding row.

## Figure S8 *Distribution of UIDS by Grouping and Latent Constructs – Extra Language Groupings Only, Broadband Domains*


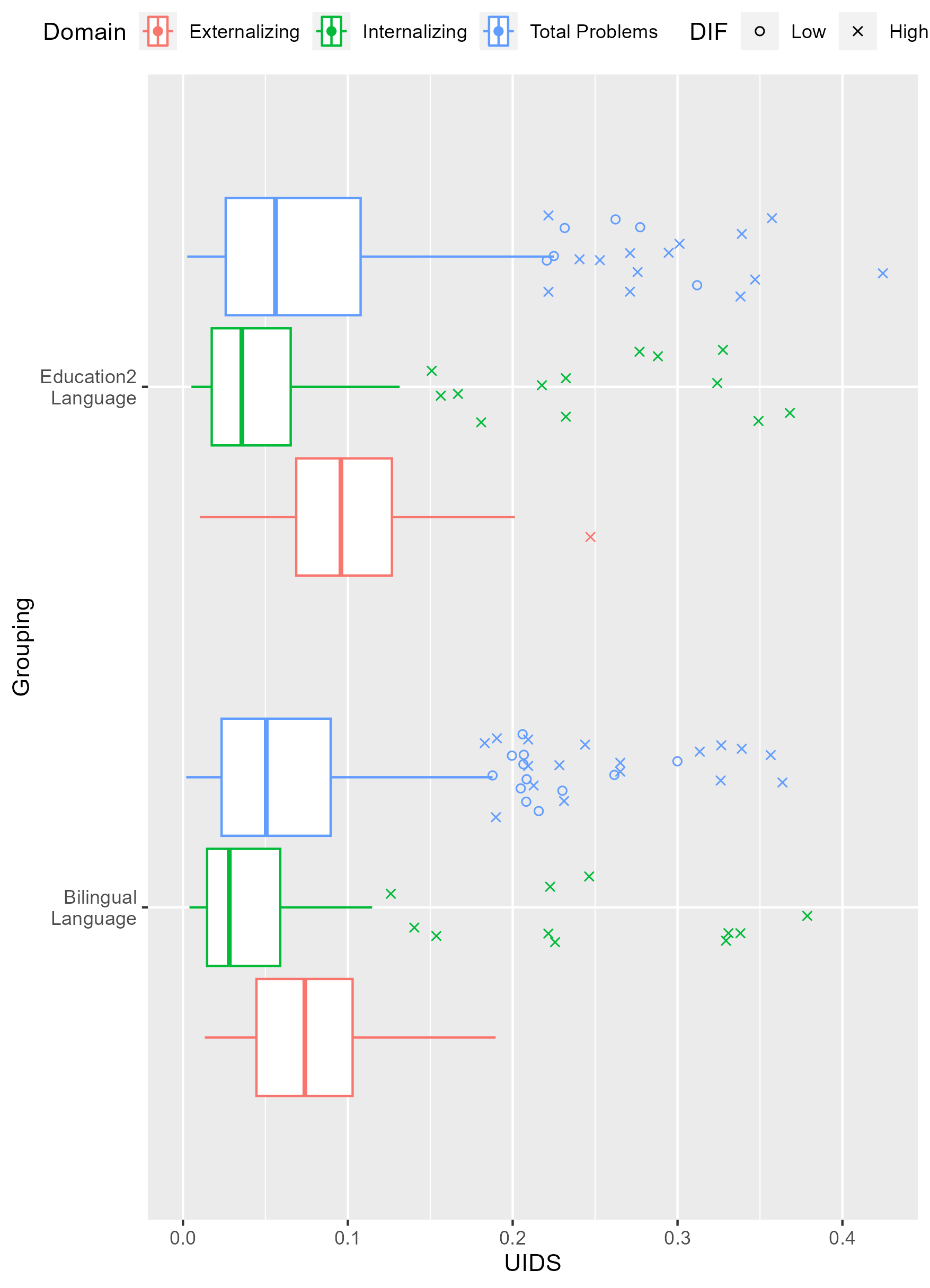


*Note*: *CareDep55*= Caregiver Depression with a cut-off of T-score>=55 for clinical range. Outliers marked with X’s correspond to UIDS from high-DIF items (Figure 2), while items marked with circles are from the robust item set (Table 3).

## Figure S9 *Distribution of UIDS by Grouping and Latent Constructs – Manuscript Groupings, Syndrome Scales*


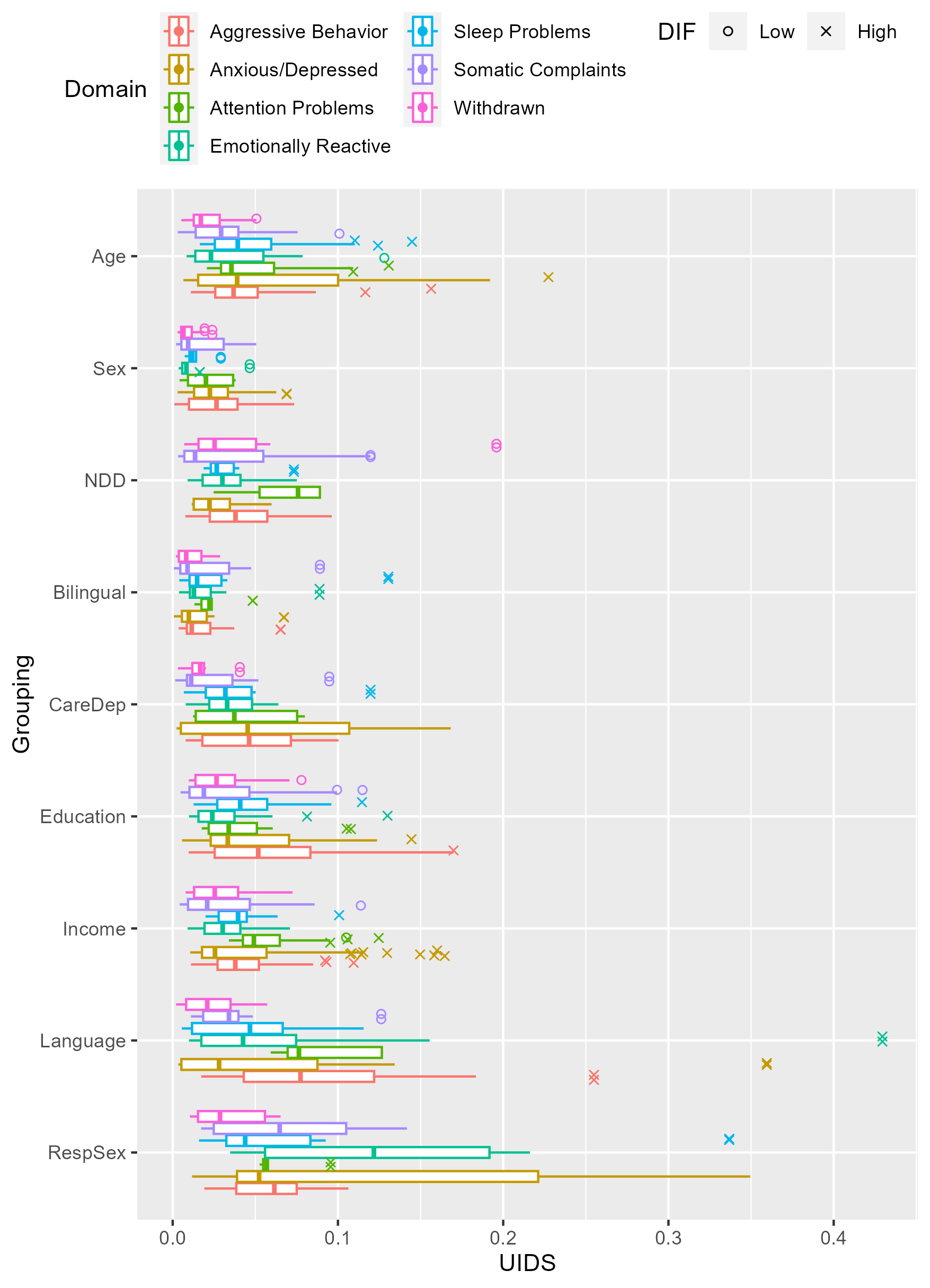


*Note*: *NDD*= Any Neurodevelopment Disorders (i.e., autism spectrum disorder, intellectual/developmental disorders, attention deficit disorder or attention deficit hyperactivity disorder, learning disability, and speech disorder; *CareDep*= Caregiver Depression with a cut-off of T-score>=60 for clinical range; *RespSex*=Respondent Sex. Outliers marked with X’s correspond to UIDS from high-DIF items (Figure 2), while items marked with circles are from the robust item set (Table 3).

## Figure S10 *Distribution of UIDS by Grouping and Latent Constructs – Extra Language Groupings Only, Syndrome Scales*


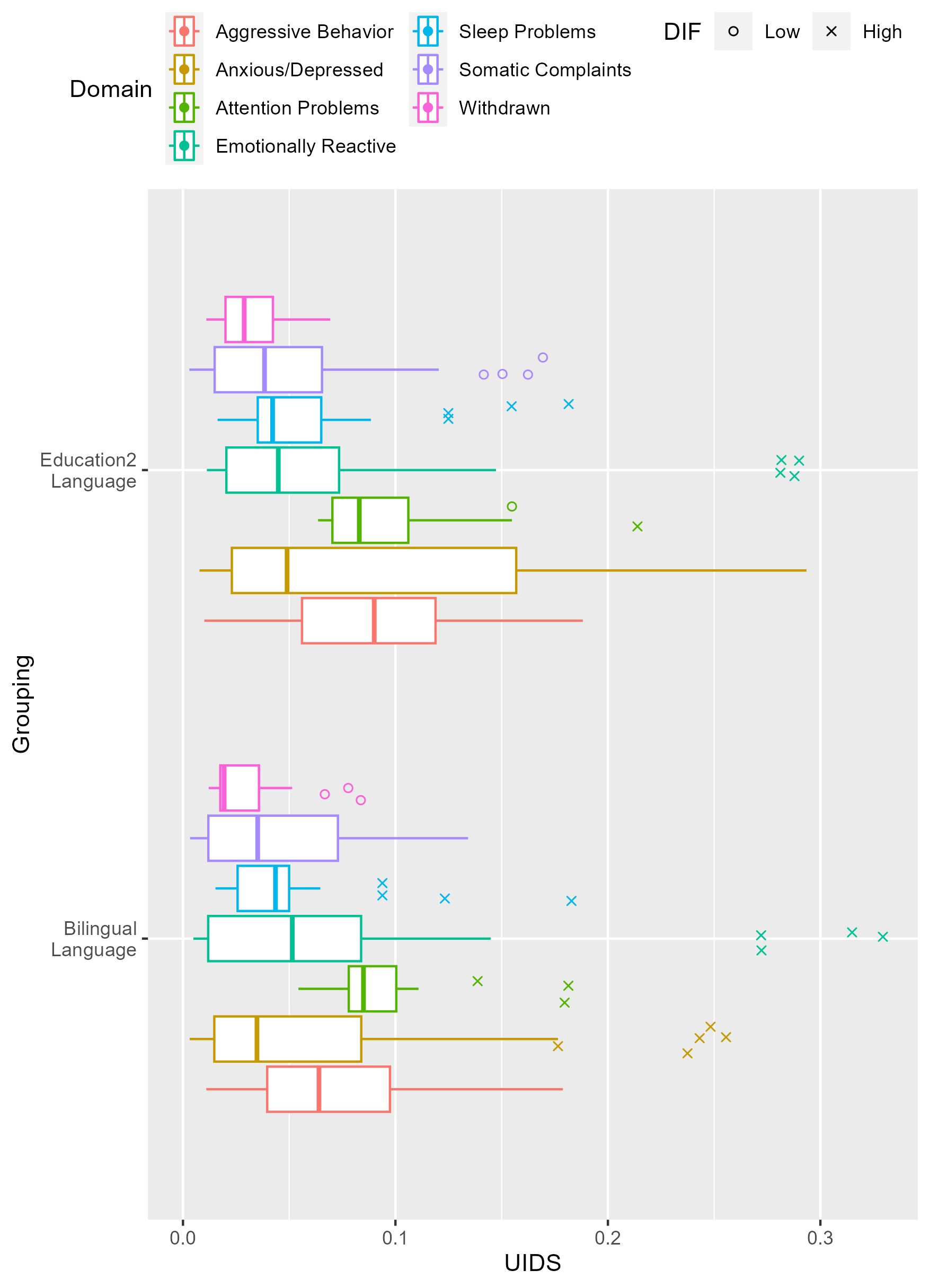


*Note*: *CareDep55*= Caregiver Depression with a cut-off of T-score>=55 for clinical range. Outliers marked with X’s correspond to UIDS from high-DIF items (Figure 2), while items marked with circles are from the robust item set (Table 3).
